# Circulating white blood cells and lung function impairment: the observational studies and Mendelian randomization analysis

**DOI:** 10.1080/07853890.2021.1948603

**Published:** 2021-07-14

**Authors:** Xiulong Wu, Chenming Wang, Hang Li, Hua Meng, Jiali Jie, Ming Fu, Yansen Bai, Guyanan Li, Wei Wei, Yue Feng, Mengying Li, Xin Guan, Meian He, Xiaomin Zhang, Huan Guo

**Affiliations:** Department of Occupational and Environmental Health, State Key Laboratory of Environmental Health (Incubating), School of Public Health, Tongji Medical College, Huazhong University of Science and Technology, Wuhan, China

**Keywords:** Lung function, white blood cell counts, LASSO regression, epidemiology, Mendelian randomization

## Abstract

**Background:**

Circulating white blood cell (WBC) counts have been related to lung function impairment, but causal relationship was not established. We aimed to evaluate independent effects and causal relationships of WBC subtypes with lung function.

**Methods:**

The 19,159 participants from NHANES 2011–2012 (*n* = 3570), coke-oven workers (COW, *n* = 1762) and Dongfeng-Tongji (DFTJ, *n* = 13,827) cohorts were included in the observational studies. The associations between circulating counts of WBC subtypes and prebronchodilator lung function were evaluated by linear regression models and LASSO regression was used to select effective WBC subtypes. Summary statistics for WBC-associated SNPs were extracted from literature, and Mendelian randomization (MR) analysis with inverse-variance weighted (IVW) method was applied to estimate the causal effects of total WBC and subtypes on lung function among 4012 subjects from COW (*n* = 1126) and DFTJ cohorts (*n* = 2886).

**Results:**

Total WBC counts were negatively associated with lung function among three populations and their pooled analysis indicated that per 1 × 10^9^ cells/L increase in total WBC was associated with 36.13 (95% CI: 30.35, 41.91) mL and 25.23 (95% CI: 19.97, 30.50) mL decrease in FVC and FEV_1_, respectively. Independent associations with lung function were found for neutrophils, monocytes, eosinophils and basophils (all *p* < .05), except lymphocytes. Besides, IVW MR analysis showed that genetically predicted total WBC and neutrophil counts were associated with reduced FVC (*p* = .017 and .021, respectively) and FEV_1_ (*p* = .048 and .043, respectively).

**Conclusions:**

WBC subtypes were independently associated with lower lung function except lymphocytes. Our findings suggest that circulating neutrophils may be causal factors in lung function impairment.

**KEY MESSAGES**
- White blood cell (WBC) subtypes were negatively associated with lung function level except lymphocytes in the observational studies.
- Associations of WBC subtypes with lung function may be modified by sex and smoking.
- Mendelian randomization analysis shows that neutrophils may be causal factors in lung function impairment.

## Introduction

Lung function reflects respiratory health and it is used in the clinical diagnosis of many lung diseases, including the chronic obstructive pulmonary disease (COPD) and asthma [1]. Circulating white blood cell (WBC) counts are widely accepted biomarkers of systematic inflammation and may play a pathological role in the process of pulmonary injury [2,3]. Circulating WBC was mainly comprised of five subtypes, including neutrophils, monocytes, lymphocytes, eosinophils and basophils. Some observational studies have evaluated the associations between WBC subtypes and lung function [4–6], but different WBC subtypes may play distinct roles in the process of lung function deterioration. The counts of total WBC, neutrophil, monocyte, lymphocyte and eosinophil were reported to be associated with lower lung function [4–6]; but there were inconsistent findings on the effect of basophils [6,7]. The associations of different WBC subtypes with lung function may be confounded by the correlations between each other [8].

Mendelian randomization (MR) study has been widely used to make causal inference between risk factors and health outcomes by using genetic variables as instrumental variables (IVs) [9]. Genetic allele is randomly transferred from parent to offspring during gamete formation, which is less susceptible to confounding and reverse causality and regarded as the nature’s randomized trial [10,11]. IVs used in the MR study should fulfil three essential conditions: (1) not associated with confounders; (2) strongly associated with exposure of interest; (3) associations of IVs with outcomes are only through the exposure of interest, not other pathways [12]. Genome-wide association studies (GWAS) have reported many significant single-nucleotide polymorphisms (SNPs) associated with circulating counts of total WBC and subtypes [8,13–15]; however, the causal relationships between WBC subtypes and lung function levels were scarcely investigated.

In this research, we first carried out three observational studies of 19,159 participants from NHANES 2011–2012 (*n* = 3570), occupational cohort of coke-oven workers (COW, *n* = 1762) and Dongfeng-Tongji (DFTJ, *n* = 13,827) cohort, aimed to evaluate the independent associations of total and differential WBC counts with lung function levels. The further two-sample MR analysis, using SNPs derived from the largest GWAS of WBC in the Asian population as IVs [8], was then performed to infer the causal associations of total WBC and subtypes with lung function among 4012 participants from COW (*n* = 1126) and DFTJ (*n* = 2886) studies.

## Methods

### Study population

NHANES 2011–2012 was a cross-sectional survey to evaluate the health and nutritional status of Americans with multistage design and oversample of Asian Americans [16], 9756 subjects were enrolled in this cycle. In the 2014, 1886 workers were enrolled from a coke-oven plant located in Wuhan, Hubei, China. Besides, in the 2013, 38,295 employees were enrolled in the DFTJ cohort from Dongfeng Motor Corporation sited in Shiyan, Hubei, China [17]. For participants from three studies, their demographic information (age, sex, race, etc.) and lifestyles (e.g. cigarette use, alcohol drinking, drug use, exercising status, medical condition and disease history) were collected by face-to-face interview using a standardized questionnaire. The detailed definitions of cigarette use, alcohol drinking and exercise were provided in the Supplementary Methods. Health examination data, including height and spirometry, were also measured. Furthermore, study participants provided blood and urine samples for laboratory determinations.

**Figure 1.**
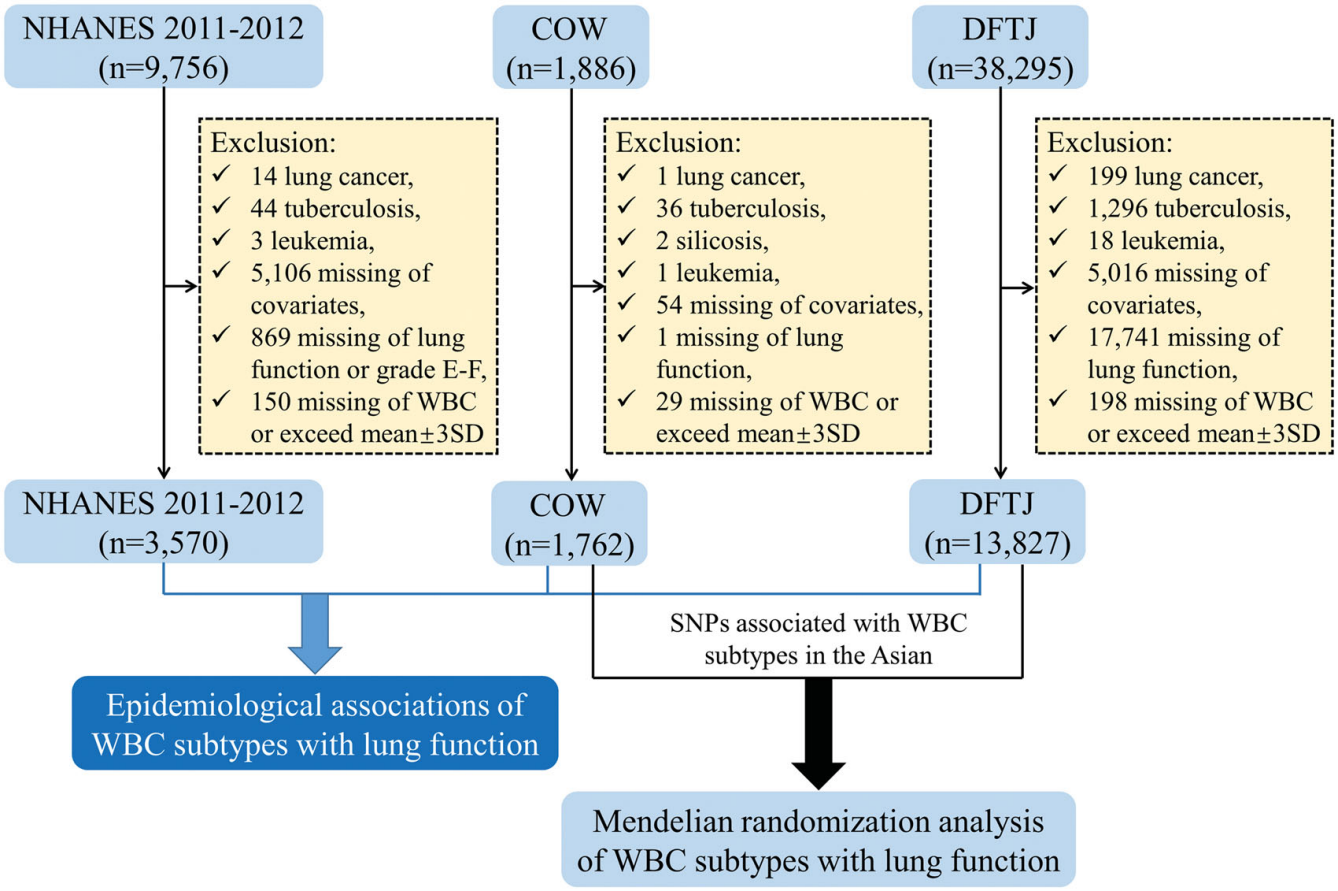
Flowchart of study design. NHANES 2011–2012: National Health and Nutrition Examination Survey 2011–2012; COW: coke-oven workers cohort; DFTJ: Dongfeng-Tongji cohort; WBC: white blood cell; SD: standard deviation.

After excluding participants with lung cancer, tuberculosis, silicosis or leukaemia, missing of covariates (age, sex, race, height, cigarette smoking, alcohol drinking and physical activity), WBC counts or lung function among the above three studies, 19,159 participants were included in the following analyses (Figure 1). All the participants gave written informed consent. NHANES 2011–2012 was approved by NCHS Research Ethics Review Board (no. 2011-17), and COW and DFTJ cohort studies were approved by the Ethics and Human Subject Committee of Tongji Medical College, Huazhong University of Science and Technology (nos. S320 and S335).

### Measurement of lung function

In the NHANES 2011–2012, trained technician used Ohio 822/827 dry-rolling seal volume spirometer (Ohio Medical Products, Wisconsin, USA) to measure lung function, and three acceptable curves were obtained from each participant. Participants with lung function graded A–C were used in the following analysis as recommended [18]. In the COW and DFTJ studies, the Chestgraph HI-101 (CHEST Ltd., Tokyo, Japan) was used to measure prebronchodilator lung function in triplicate, including FVC and FEV_1_; results were recorded following the guidelines from ATS/ERS Task Force [19].

### Determinations of total and differential WBC counts

In the NHANES 2011–2012, Beckman Coulter HMX Hematology Analyser (Beckman Coulter, Brea, CA) was used to count the number of circulating total and five differential WBC subtypes. In the COW study, Sysmex XT-4000i (Sysmex Corporation, Kobe, Japan) was applied to perform 3-Diff analysis of WBC, including total WBC, neutrophils, lymphocytes and intermediate cell (sum of monocytes, eosinophils and basophils). In the DFTJ study, Architect Ci8200 integrated system (Abbott Laboratories, Abbott Park, IL) was used to measure circulating total and five differential WBC subtypes following the instruction of manufacture. Subjects with total WBC counts exceeded three standard deviations from the mean were excluded.

### Genotyping and selections of IVs

Among the study subjects, the genotyping results of participants from NHANES 2011–2012 were unavailable in the public dataset; the 1126 participants from COW cohort were genotyped by using Illumina Infinium Global Screening Array-24 MD BeadChip and 2886 participants from DFTJ cohort were genotyped by using Affymetrix Genome-Wide Human SNP Array (*n* = 478) and Illumina Infinium OmniZhongHua-8 BeadChip (*n* = 2408). These 4012 Chinese subjects were included in the further MR analysis. The integrated release version 5 haplotypes of all 2504 samples in the 1000 Genomes Project Phase 3 was used as reference panel and Eagle 2.3.5 and Minimac3 were applied for SNPs imputation.

For genetic associations with total and differential WBC counts, we used summary data from the most up-to-date and largest GWAS in the Asian population, which has reported 36, 21, 31, 18 and 26 SNPs associated with the blood counts of total WBC, neutrophil, monocyte, eosinophil and basophil, respectively [8]. Trait-specific SNPs locate at a distance of ≥1 Mb apart from each other, and they are not in linkage disequilibrium in the East Asians (*r*^2^ < 0.1). The *F* statistics were calculated to evaluate the strength of the associations, and SNPs with *F* statistic values <10 were considered as weak IVs [20].

### Statistical analysis

The distributions of eosinophils and basophils were right skewed, and they were log10-transformed to approximate normal distribution; other continuous variables were in normal distribution. In the single-marker model, the counts of total and differential WBC were separately entered as an independent variable to evaluate their individual associations with FVC and FEV_1_, with adjustment for age, sex, race (only in NHANES 2011–2012 population), height, smoking status, alcohol use and exercise (these variables were considered as covariates unless otherwise specified). The complex design was considered for the NHANES 2011–2012 data in the analyses, including strata, cluster and sample weight [21]. Results from three populations were pooled by meta-analysis using fixed-effect (heterogeneity *p* ≥ .05) or random-effect (heterogeneity *p* < .05) method. Restricted cubic spline (RCS) plots were used to explore the shapes of the above associations. Sensitivity analysis was performed by excluding subjects with cancers or using anti-infectious drugs (information on drug use was only available in the NHANES 2011–2012 and DFTJ cohort).

For the different WBC subtypes those had significant associations with lung function in the single-marker model (*p* < .05), we further used least absolute shrinkage and selection operator (LASSO) regression with 10-fold cross-validation to select the most significant WBC subtypes at minimum mean squared error, and covariates were entered into the LASSO regression without penalization [22]. The selected WBC subtypes were then simultaneously included in the multiple-marker model, with adjustment for the same covariates used in the single-marker model.

Besides, the interaction effects of WBC counts with sex and smoking were also investigated in the three populations, and *P*_int_ was obtained by entering a cross-product term in the model (WBC × sex or WBC × smoking) when total and differential WBC counts were separately entered into the model.

Furthermore, two-sample MR analysis was used to evaluate the causal relationships of total and differential WBC with lung function. Summary statistics of WBC-associated SNPs were extracted from the largest GWAS in the Japanese population [8]. The SNPs associated with confounding covariates (height, smoking status, alcohol use and exercise) at the Bonferroni-corrected significance level were excluded from IVs. The primary method we used to perform MR analysis was the inverse-variance weighted (IVW) estimation [23]. The MR-PRESSO and MR-Egger regression were used to evaluate horizontal pleiotropy and potential outliers were excluded [24,25]. The SAS 9.4 (SAS Inc., Cary, NC), Stata/SE 12.0 (StataCorp, College Station, TX) and R 3.5.3 software (Vienna, Austria) were used in the statistical analysis and a two-sided *p* < .05 was defined as statistical significance unless otherwise specified. SAS (SAS Inc., Cary, NC) and R codes (Vienna, Austria) of main results were provided in the Supplementary Notes.

## Results

### Characteristics of study participants

The general information for subjects in the NHANES 2011–2012 (*n* = 3570), COW (*n* = 1762) and DFTJ (*n* = 13,827) cohorts is shown in Table 1. Among the three populations, the mean age (standard error, SE) was 45.43 (0.79), 41.33 (0.22) and 64.40 (0.07) years-old, and the proportion of male subjects was 49.6%, 87.1% and 43.2%, respectively (Table 1). Besides, smoking rate was 43.8%, 58.2% and 27.3% in the NHANES 2011–2012, COW and DFTJ cohorts, respectively.

**Table 1.** General characteristics of study participants.

Variables	NHANES 2011–2012 (*n* = 3570)	COW (*n* = 1762)	DFTJ (*n* = 13,827)
Age, years	45.43 (0.79)	41.33 (0.22)	64.40 (0.07)
Sex			
Males	1821 (49.6)	1535 (87.1)	5972 (43.2)
Females	1749 (50.4)	227 (12.9)	7855 (56.8)
Race			
Non-Hispanic Asian	463 (4.4)	1762 (100.0)	13,827 (100.0)
Mexican American	356 (7.4)	−	−
Other Hispanic	360 (6.1)	−	−
Non-Hispanic White	1334 (68.6)	−	−
Non-Hispanic Black	944 (10.8)	−	−
Other race	113 (2.7)	−	−
Height, cm	169.28 (0.27)	170.12 (0.15)	160.07 (0.07)
BMI, kg/m^2^	28.86 (0.24)	24.01 (0.08)	24.31 (0.03)
Smoking status			
Non-smokers	2039 (56.2)	736 (41.8)	10,049 (72.7)
Smokers	1531 (43.8)	1026 (58.2)	3778 (27.3)
Alcohol use			
Non-drinkers	451 (8.8)	1105 (62.7)	9559 (69.1)
Drinkers	3119 (91.2)	657 (37.3)	4268 (30.9)
Exercising status			
Non-exercisers	2942 (80.5)	721 (40.9)	1351 (9.8)
Exercisers	628 (19.5)	1041 (59.1)	12,476 (90.2)
Total and differential WBC counts, ×10^9^/L			
Total WBC	6.93 (0.09)	6.36 (0.03)	5.38 (0.01)
Neutrophils	4.12 (0.06)	3.60 (0.02)	3.25 (0.01)
Lymphocytes	2.04 (0.02)	2.18 (0.01)	1.59 (0.004)
Monocytes	0.51 (0.01)	−	0.28 (0.001)
Eosinophils	0.16 (0.05 0.43)	−	0.09 (0.03 0.32)
Basophils	0.04 (0.01 0.13)	−	0.06 (0.02 0.20)
Lung function parameters			
FVC, mL	4112.97 (26.72)	3488.55 (17.61)	2422.85 (5.96)
FEV_1_, mL	3207.40 (27.43)	3108.48 (15.10)	2080.33 (5.10)
FEV_1_/FVC, %	77.92 (0.36)	89.53 (0.17)	86.38 (0.09)

NHANES 2011–2012: National Health and Nutrition Examination Survey 2011–2012; COW: coke-oven workers cohort; DFTJ: Dongfeng-Tongji cohort; BMI: body mass index; WBC: white blood cell; FVC: forced vital capacity; FEV_1_: forced expiratory volume in one second.

Values were presented as mean (SE) or median (5^th^, 95^th^ percentiles) for continuous variables and *n* (%) for categorical variables. In the NHANES 2011–2012 study, exam weight was taken into account.

### Relationships of total and differential WBC counts with lung function

**Figure 2.**
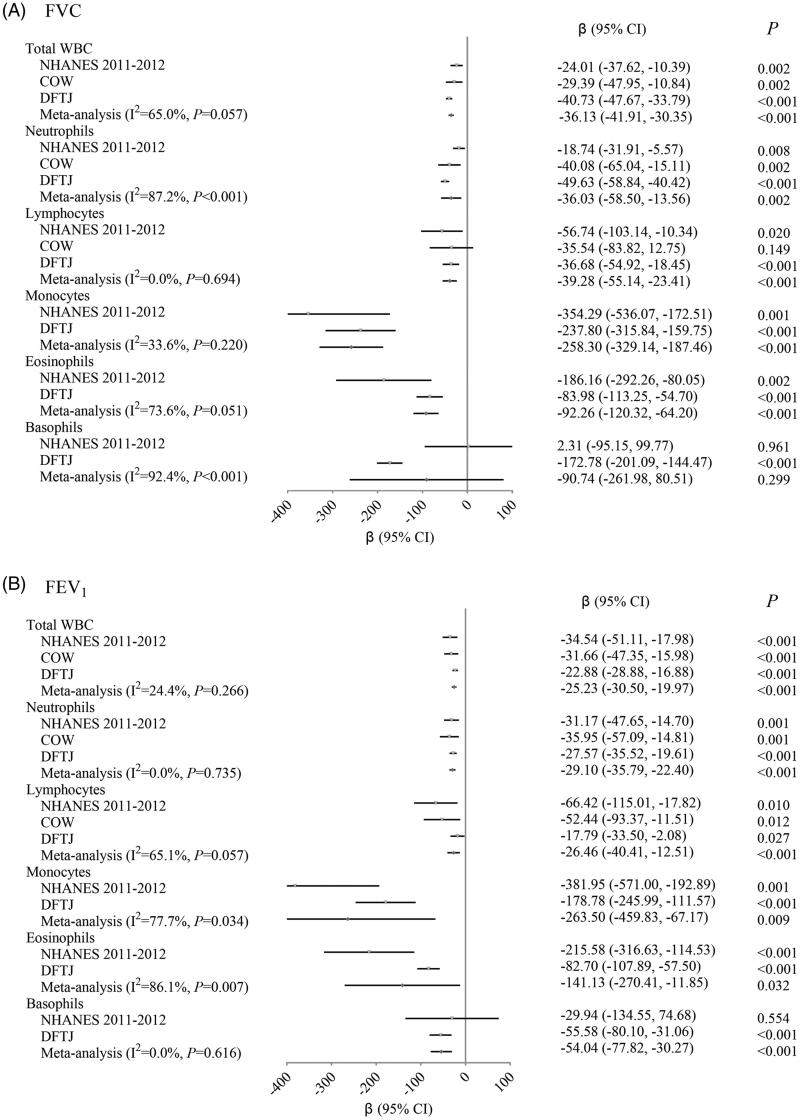
Relationships of total and differential WBC counts with FVC (A) and FEV_1_ (B) (single-marker model). NHANES 2011–2012: National Health and Nutrition Examination Survey 2011–2012; COW: coke-oven workers cohort; DFTJ: Dongfeng-Tongji cohort; WBC: white blood cell; FVC: forced vital capacity; FEV_1_: forced expiratory volume in one second. Total and differential WBC counts were separately included in the multiple linear regression model, and the model was adjusted for age, sex, race (only in NHANES 2011–2012 population), height, smoking, alcohol use and exercise. Eosinophil and basophil counts were transformed by common logarithm (log10) to approximate normal distribution. Fixed-effect (heterogeneity *p*≥.05) or random-effect (heterogeneity *p*<.05) meta-analysis was used to combine results from three studies.

Total WBC were well correlated with neutrophils, and modestly with lymphocytes and monocytes (Table S1). The meta-analysis revealed that per 1 × 10^9^ cells/L increase in the total WBC was associated with a separate 36.13 (95% CI: 30.35, 41.91) mL and 25.23 (95% CI: 19.97, 30.50) mL decrease in FVC (Figure 2(A)) and FEV_1_ (Figure 2(B)); and there were consistently negative associations of total WBC with FVC and FEV_1_ among the NHANES 2011–2012, COW and DFTJ studies (Figure 2). In the single-marker model, neutrophils, lymphocytes, monocytes and eosinophils all showed negative associations with FVC and FEV_1_ in the meta-analysis (all *p* < .05), as well as basophils–FEV_1_ association (*p* < .001, Figure 2). In the COW study, intermediate cell counts were also significantly associated with FVC and FEV_1_ in the single-marker analysis (data not shown). RCS plots further confirmed their linear dose–response relationships except lymphocytes–FVC association in the COW population (Figures S1–S6). The sensitivity analysis among subjects without cancers or without using anti-infectious drugs also confirmed the concordant directions and similar effect sizes (Tables S2–S3). In the multiple-marker model, we observed the independent associations of neutrophils and basophils with FVC, and of neutrophils, monocytes, eosinophils and basophils with FEV_1_ (all *p* < .05, Table 2). Lymphocytes were no longer significantly associated with FVC or FEV_1_ in the multiple-marker model (*p* = .234 and .748, respectively; Table 2).

**Table 2.** Relationships of differential white blood cell counts with FVC and FEV_1_ selected by LASSO regression (multiple-marker model).

WBC subtypes	FVC, mL		FEV_1_, mL	
	*β* (95% CI)	*p*	*β* (95% CI)	*p*
*NHANES 2011–2012 (n = 3570)*				
Neutrophils	−	−	−16.56 (−33.32, 0.20)	.052
Lymphocytes	−	−	−12.01 (−59.83, 35.81)	.603
Monocytes	−314.48 (−506.82, −122.13)	.003	−264.42 (−475.60, −53.24)	.017
Eosinophils	−149.62 (−257.54, −41.70)	.009	−170.07 (−266.08, −74.07)	.002
*COW (n = 1762)*				
Neutrophils	−26.05 (−53.26, 1.17)	.061	−	−
*DFTJ (n = 13,827)*				
Neutrophils	−33.65 (−43.96, −23.34)	<.001	−20.13 (−29.05, −11.22)	<.001
Lymphocytes	−11.70 (−30.98, 7.58)	.234	4.61 (−12.05, 21.28)	.588
Monocytes	−108.20 (−195.75, −20.65)	.015	−97.05 (−172.72, −21.38)	.012
Eosinophils	−31.24 (−62.28, −0.19)	.049	−64.95 (−91.78, −38.11)	<.001
Basophils	−143.55 (−173.34, −113.77)	<.001	−28.56 (−54.30, −2.82)	.030
*Meta-analysis (n = 19,159)*				
Neutrophils	−32.69 (−42.34, −23.05)	<.001	−19.25 (−26.99, −11.52)	<.001
Heterogeneity *p*	.608		.697	
Lymphocytes	−11.70 (−30.98, 7.58)	.234	2.56 (−13.04, 18.16)	.748
Heterogeneity *p*	1.000		.492	
Monocytes	−196.03 (−395.94, 3.89)	.055	−118.72 (−189.32, −48.13)	.001
Heterogeneity *p*	.042		.119	
Eosinophils	−80.44 (−194.78, 33.91)	.168	−108.55 (−210.07, −7.04)	.036
Heterogeneity *p*	.027		.027	
Basophils	−143.55 (−173.34, −113.77)	<.001	−28.56 (−54.30, −2.82)	.030
Heterogeneity *p*	1.000		1.000	

NHANES 2011–2012: National Health and Nutrition Examination Survey 2011–2012; COW: coke-oven workers cohort; DFTJ: Dongfeng-Tongji cohort; FVC: forced vital capacity; FEV_1_: forced expiratory volume in one second.

WBC subtypes selected by LASSO regression were included in the multiple linear regression model simultaneously, and the model was adjusted for age, sex, race (only in NHANES 2011–2012 population), height, smoking, alcohol use and exercise. Eosinophil and basophil counts were transformed by common logarithm (log10) to approximate normal distribution. Specifically, in the COW study, neutrophils and intermediate cell counts (data no shown) were selected by LASSO regression and both of them were included in the multiple-marker regression analysis for FVC. Fixed-effect (heterogeneity *p*≥ .05) or random-effect (heterogeneity *p*< .05) meta-analysis was used to combine results from three studies.

**Figure 3.**
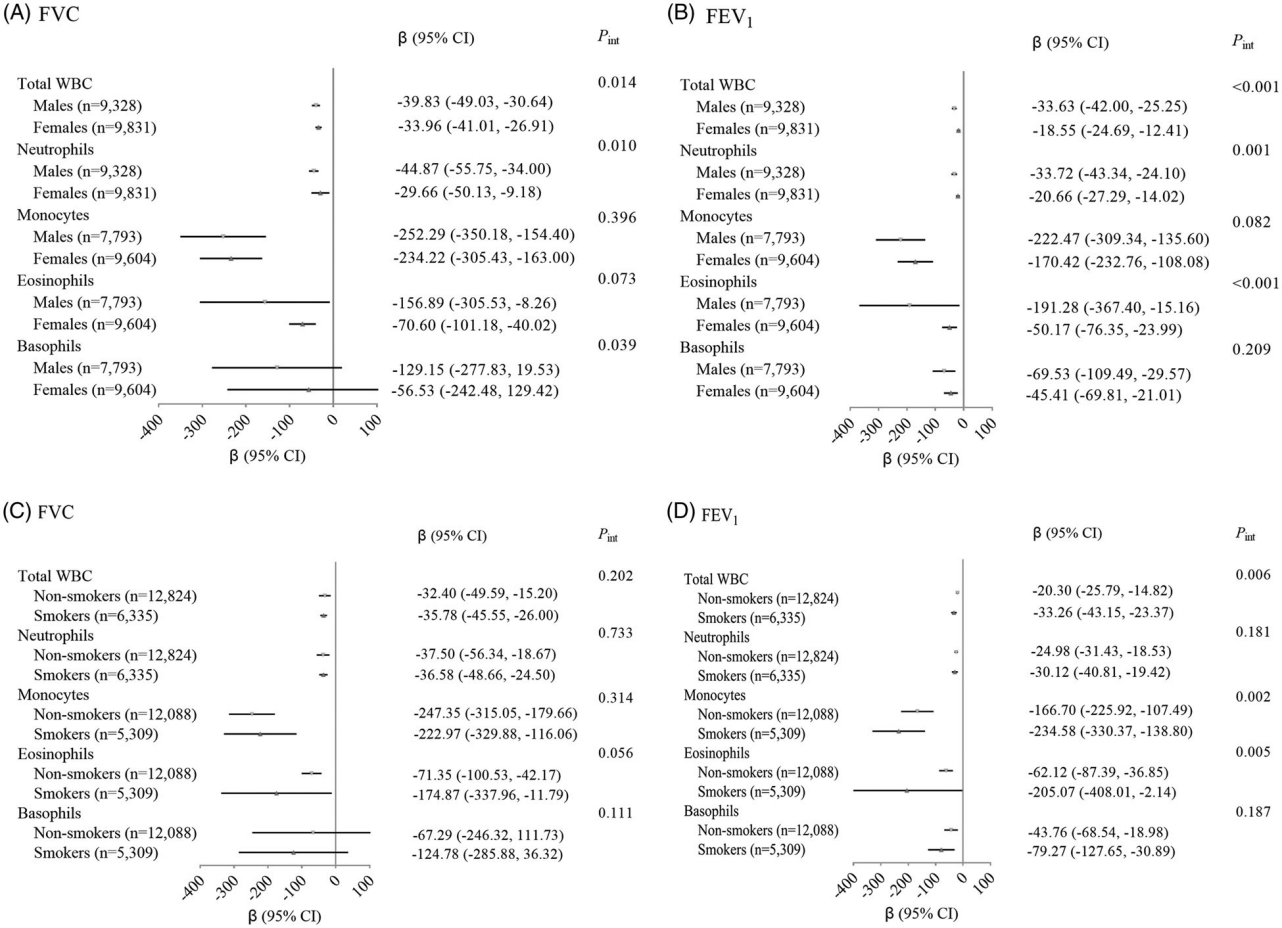
Interaction effects of total and differential WBC counts with sex and smoking on FVC and FEV_1_ in the meta-analysis of three populations. (A) Interaction effects of total and differential WBC counts with sex on FVC. (B) Interaction effects of total and differential WBC counts with sex on FEV_1_. (C) Interaction effects of total and differential WBC counts with smoking on FVC. (D) Interaction effects of total and differential WBC counts with smoking on FEV_1_. WBC: white blood cell; FVC: forced vital capacity; FEV_1_: forced expiratory volume in one second. Fixed-effect (heterogeneity *p*≥.05) or random-effect (heterogeneity *p*<.05) meta-analysis was used to combine results from three studies.

The effects of total WBC on decreasing FVC and FEV_1_ were enhanced among males than females (*P*_int_=0.014 and <0.001 in the meta-analysis of three populations, respectively; Figure 3(A,B)). The significant interactions with sex were also observed for neutrophils on FVC (*P*_int_=0.010) and FEV_1_ (*P*_int_=0.001), but not for monocytes (*P*_int_=0.396 and 0.082, respectively). Besides, there was significant interaction of eosinophils with sex on FEV_1_ (*P*_int_ < 0.001) and interaction of basophils with sex on FVC (*P*_int_=0.039). When stratified by smoking status, we did not observe the modification effect of smoking on the associations of total WBC and WBC subtypes with FVC (all *P*_int_>0.05 in the meta-analysis, Figure 3(C)). However, the meta-analysis indicated significant modification effect of smoking on the association between total WBC and FEV_1_, which was stronger in the smokers than that in the non-smokers (smokers: *β* (95% CI)= −33.26 (−43.15, −23.37) vs. non-smokers: *β* (95% CI)= −20.30 (−25.79, −14.82), *P*_int_=0.006; Figure 3(D)). As for the WBC subtypes, smoking could significantly modify the associations of neutrophils (only in NHANES 2011–2012, *P*_int_=0.048), monocytes (meta *P*_int_=0.002) and eosinophils (meta *P*_int_=0.005) with FEV_1_, but not for basophils (meta *P*_int_=0.187).

### MR analyses

Among the reported SNPs associated with total and differential WBC counts, the 33, 19, 30, 17 and 21 SNPs were separately included as candidate IVs in the MR analysis of total WBC, neutrophil, monocyte, eosinophil and basophil counts with lung function, respectively, after excluding those SNPs without genotyping information, with *F* statistics < 10 (Table S4), associated with confounders, or potential outliers flagged up by MR-PRESSO method (Table S5). The MR-PRESSO global test did not detect the existence of horizontal pleiotropy of these IVs and the intercept of MR-Egger regression did not deviate from 0 (all *p*> .05). As shown in Table S6, we did not find any between-instrument heterogeneity for IVW and MR-Egger methods in the MR analysis (all heterogeneity *p*> .05). The IVW method suggested that genetically determined total WBC counts were significantly associated with reduced FVC (*β* (95% CI)= −145.12 (−263.81, −26.43), *p* = .017) and FEV_1_ (*β* (95% CI)= −103.87 (−206.97, −0.77), *p* = .048). Then, an inverse causal association with FVC and FEV_1_ was shown for neutrophil counts (IVW, *β* (95% CI)= −131.90 (−243.90, −19.90) and −105.10 (−207.11, −3.09), and *p* = .021 and .043, respectively), but not for monocyte, eosinophil or basophil counts (all IVW *p*> .05, Table 3). Results from MR-Egger method also indicate marginally significant associations of genetically determined total WBC and neutrophil counts with decreased FVC and FEV_1_ (Table 3).

**Table 3.** Mendelian randomization analyses for the causality between total and differential WBC counts and lung function.

Total and differential WBC counts	No. of SNPs	MR method	FVC, mL		FEV_1_, mL	
			*β* (95% CI)	*p*	*β* (95% CI)	*p*
Total WBC	33	IVW	−145.12 (−263.81, −26.43)	.017	−103.87 (−206.97, −0.77)	.048
		MR-Egger estimate	−314.61 (−652.19, 22.97)	.068	−273.15 (−566.35, 20.06)	.068
		MR-Egger intercept	7.31 (−6.32, 20.95)	.293	7.30 (−4.54, 19.15)	.227
		MR-PRESSO global test *p*		.798		.620
Neutrophils	19	IVW	−131.90 (−243.90, −19.90)	.021	−105.10 (−207.11, −3.09)	.043
		MR-Egger estimate	−307.37 (−637.24, 22.51)	.068	−293.86 (−589.86, 2.13)	.052
		MR-Egger intercept	10.83 (−8.33, 29.98)	.268	11.64 (−5.54, 28.83)	.184
		MR-PRESSO global test *p*		.381		.239
Monocytes	30	IVW	−72.86 (−170.30, 24.58)	.143	−75.71 (−160.38, 8.96)	.080
		MR-Egger estimate	64.72 (−282.60, 412.05)	.715	59.72 (-241.94, 361.38)	.698
		MR-Egger intercept	−7.27 (−24.88, 10.35)	.419	−7.16 (−22.46, 8.14)	.359
		MR-PRESSO global test *p*		.473		.701
Eosinophils	17	IVW	−18.73 (−141.63, 104.17)	.765	−2.19 (−101.63, 97.25)	.966
		MR-Egger estimate	32.99 (−287.16, 353.14)	.840	47.57 (−204.83, 299.97)	.712
		MR-Egger intercept	−3.26 (−21.83, 15.30)	.730	−3.14 (−17.75, 11.48)	.674
		MR-PRESSO global test *p*		.336		.634
Basophils	21	IVW	−31.44 (−152.18, 89.31)	.610	−35.75 (−133.88, 62.37)	.475
		MR-Egger estimate	34.70 (−234.53, 303.93)	.801	22.95 (−195.36, 241.25)	.837
		MR-Egger intercept	−4.57 (−21.14, 11.99)	.588	−4.06 (−17.50, 9.37)	.554
		MR-PRESSO global test *p*		.085		.167

WBC: white blood cell; FVC: forced vital capacity; FEV_1_: forced expiratory volume in one second; IVW: inverse-variance weighted; MR: Mendelian randomization.

In the GWAS of total WBC counts, 107,964 subjects were included; and 62,076 subjects were included in the GWAS of neutrophil, monocyte, eosinophil and basophil counts [8]. In the association analyses of WBC-associated SNPs with FVC and FEV_1_, 4012 participants from COW and DFTJ studies were included.

## Discussion

We conducted a multi-centre study to investigate the relationships of WBC subtypes with lung function using integration of multiple-marker approach and genetic data. Results from multiple-marker model showed that elevated levels of total and major WBC subtypes (except lymphocytes) were independently associated with decreased FVC and FEV_1_. We observed that associations of total WBC, neutrophil, monocyte, eosinophil and basophil counts with lung function may be modified by sex and tobacco smoking when WBC subtypes were separately included in the regression analysis. Furthermore, the MR analysis indicated the causal relationships of elevated total WBC and neutrophil counts with FVC and FEV_1_ reduction among the Asian populations.

In previous reported NHANES III study, total WBC was negatively associated with FVC and FEV_1_; participants in the highest quintile of neutrophils and monocytes had lower levels of FVC and FEV_1_ than those in the lowest quintile [6]. A 15-year follow up study of 9434 firefighters, who had joined in the World Trade Center disaster rescue, showed that increased blood counts of neutrophil and eosinophil were related to accelerated decline in FEV_1_ [5]. The negative associations of neutrophil, monocyte and eosinophil counts with FVC and FEV_1_ were also observed in the present research. Consistent with previous findings in NHANES III study [6], we found that the associations of lymphocytes with FVC and FEV_1_ were significant in the single-marker model but abolished after adjustment for other WBC subtypes in the multiple-marker model. Similarly, Calciano et al. did not find the significant association between blood lymphocytes and FEV_1_/FVC when eosinophil and basophil counts were simultaneously included in the same model for mutual adjustment [26]. The NHANES III study did not observe a significant relationship of basophil counts with either FVC or FEV_1_ [6]. In our results, the inverse association of basophil counts with lung function was also not observed in individuals from NHANES 2011–2012, but was significant among DFTJ cohort participants. Lewis et al. also reported a negative association between blood basophils and FEV_1_ in 2369 British adults [7]. The inconsistent results may due to the distinct population stratification of these studies. The independent effects of lymphocytes and basophils warranted further validations.

The mechanism underlying the negative association of WBC with lung function involved the release of proteinases by inflammatory cells. Elevated blood neutrophils and monocytes were associated with increased innate host defence in the process of bacterial infection, and eosinophils and basophils was related to type-2 inflammation. Neutrophils secret many types of proteases under the stimulation of pathogenic microorganism or environmental pollutants, including serine proteases (like neutrophil elastase and proteinases 3), matrix metalloproteinases (MMPs, such as MMP-1 and MMP-12) and cysteine proteinases (including cathepsin G) [27]. Blood monocyte is the precursor of lung macrophage, cigarette smoke exposure leads to overproduction of granulocyte-macrophage colony-stimulating factor and then macrophage activation. Activated alveolar macrophage secrets MMPs, including MMP-1, MMP-8 and MMP-12, and cathepsins K, L and S [28]. Blood eosinophil is a surrogate of eosinophilic lung inflammation, and it predicts lung function deterioration [7,29]. Activated eosinophil would produce cytotoxic proteinases, including eosinophil peroxidase and eosinophil cationic protein [28,30], and generate IL-13 to stimulate the production of MMP-12 by macrophage [31,32]. The mechanism underlying the basophil-related lung function impairment was not elucidated. IL-4 secreted by activated basophil would induce a shift from lung-infiltrating monocyte to interstitial macrophage and IL-4/macrophage/macrophage-derived MMP-12 axis plays an important role in the development of emphysema [33]. All these proteases would destroy lung parenchyma, lead to destruction of alveolar structure, airspace enlargement, lung function deterioration and emphysema in the presence of protease–antiprotease imbalance.

In the present work, we found marginal or significant interactions of neutrophils (in NHANES 2011–2012), monocytes and eosinophils with smoking on FEV_1_. Consistent with our findings, Zeig-Owens et al. reported stronger associations of blood neutrophils and eosinophils with reduced FEV_1_ in smokers than that in never-smokers (*P*_int_=0.010 and 0.004, respectively) [5]. The underlying mechanisms were still unknown. Cigarette smoke could cause direct damage of lung, including airway epithelial thickening, goblet cell hyperplasia and alveolar cell apoptosis [34,35]; smokers were reported to have higher counts of neutrophil, monocyte and eosinophil than non-smokers [36,37], which would accelerate the decrease in lung function among smokers.

MR analysis helps to evaluate the causal relationship of WBC subtypes with lung function. To our knowledge, only one MR study investigated the association of circulating eosinophil counts with FEV_1_ by using Netherlands LifeLines cohort data (*n* = 13,301) and they did not find causal association [38], which was consistent with the current results. However, we found that genetically elevated total WBC and neutrophil counts were associated with decreased FVC and FEV_1_ among the Asian population. The selected SNPs as IVs fulfilled the basic assumption of MR. First, IVs were not associated with confounders and potential outliers detected by MR-PRESSO method were also excluded. Second, IVs were derived from the largest GWAS of WBC in the Japanese and they were strongly associated with individual WBC subtype [8]. Third, the horizontal pleiotropy was not detected by MR-PRESSO and MR-Egger regression, suggesting the relationships of IVs with lung function were only through WBC. Thus, results derived from MR analysis were reliable in the present research. Given that WBC subtypes were significantly correlated with each other, thus multiple testing was not taken into account. Meanwhile, we noticed that statistical significance was relatively weak in the MR analyses of total WBC and neutrophil counts with lung function, causal inference should be done cautiously.

This study benefitted from the large population-based studies including three populations in U.S. and China, which increased the statistical power and validated the findings across the different races. However, some limitations should not be neglected. First, there were differences in the demographics of three datasets. However, we observed negative associations of circulating total WBC counts with FVC and FEV_1_ across different ancestries, especially for neutrophils. Second, two-sample MR analysis helps to assess the causal relationship between WBC and lung function, but it was only performed in a subset population from COW and DFTJ cohorts. Further MR studies in larger sample size and other ethnic populations were warranted to validate the present findings.

In conclusion, our observational results suggested that total WBC and its subtypes, except lymphocytes, were independently and negatively related to lung function. The MR analysis further confirmed the causal associations of total WBC and neutrophils with FVC and FEV_1_. Our findings highlight the causal relationship of neutrophils (rather than other WBC subtypes) with lung function and emphasize the effect of systematic inflammation in the pathogenesis of respiratory injury.

## Supplementary Material

Supplemental Material

## Data Availability

The data that support the findings of this study are available from the corresponding author, Huan Guo, upon reasonable request.
