# Supplementary material for: Circulating white blood cells and lung function impairment: the observational studies and Mendelian randomization analysis
Source: Ann Med. 2021 Jul 14;53(1):1119–29. doi: 10.1080/07853890.2021.1948603 (PMC8280897; doi:10.1080/07853890.2021.1948603)
Supplement: Supplemental Material [file IANN_A_1948603_SM3508.pdf]

Supplementary materials

Tables of Contents

SUPPLEMENTARY METHODS

SUPPLEMENTARY NOTES

Table S1 Pearson's correlation coefficients between different WBC subtypes.

Table S2 Relationships of total and differential WBC counts with lung function among subjects without cancers (single-marker model).

Table S3 Relationships of total and differential WBC counts with lung function among subjects without using anti-infectious drugs (single-marker model).

Table S4 Characteristics of SNPs associated with total and differential WBC counts in published GWAS.

Table S5 The associations of total and differential WBC counts related SNPs with confounders in the COW and DFTJ studies.

Table S6 Between-instrument heterogeneity test for the Mendelian randomization analysis of total and differential WBC counts with lung function.

Figure S1 Restricted cubic spline plot to show the relationship of total WBC counts with lung function.

Figure S2 Restricted cubic spline plot to show the relationship of neutrophil counts with lung function.

Figure S3 Restricted cubic spline plot to show the relationship of lymphocyte counts with lung function.

Figure S4 Restricted cubic spline plot to show the relationship of monocyte counts with lung function.

Figure S5 Restricted cubic spline plot to show the relationship of eosinophil counts with lung function.

Figure S6 Restricted cubic spline plot to show the relationship of basophil counts with lung function.

SUPPLEMENTARY METHODS

Definitions of common lifestyles

In the NHANES 2011-2012, subjects with a positive answer to the question "Have you smoked at least 100 cigarettes in your entire life" were classified as smokers; otherwise, they were classified as non-smokers [1]. Subjects with at least 12 alcohol drinks per year or in lifetime were classified as alcohol drinkers; otherwise, they were classified as non-alcohol drinkers [2]. Subjects with vigorous work activity ≥75 min/week or moderate work activity ≥150 min/week or a combination ≥150 min/week were classified as exercisers; otherwise, they were classified as non-exercisers [3].

In the COW and DFTJ studies, participants who have smoked >1 cigarette/day for >6 months were classified as smokers; otherwise, they were classified as non-smokers. Participants who have drunk alcohol >1 time/week for >6 months were classified as alcohol drinkers; otherwise, they were classified as non-alcohol drinkers [4]. During the last 6 months, participants who have spent ≥30 min at a time and ≥5 times/week exercising in leisure time were classified as exercisers; otherwise, they were classified as non-exercisers.

SUPPLEMENTARY NOTES

Single-marker regression, multiple-marker regression, and interaction analyses were performed with SAS 9.4, and LASSO regression and MR analyses were conducted using R 3.5.3 software. SAS and R codes were listed as follows.

1. SAS codes for single-marker regression analysis

```
/*NHANES -2012 study, analyzing code for relationship of total WBC counts with FVC was listed as an example*/
PROC SURVEYREG data=NHANES nomcar;
STRATA SDMVSTRA;
CLUSTER SDMVPSU;
WEIGHT WTMEC2YR;
CLASS race;
MODEL FVC=age sex race height smoke drink exercise WBC/clparm solution
vadjust=none;
run;
/*COW and DFTJ studies, analyzing code for relationship of total WBC counts with FVC in the COW study was listed as an example*/
PROC GLM data=COW;
MODEL FVC =age sex height smoke drink exercise WBC/clparm solution;
run;
```

2. R codes for LASSO regression analysis

```
library(glmnet)

# LASSO regression analysis in the NHANES 2011-2012 study was listed as an example

NHANES<-read.table("NHANES.txt",sep="\t",header=T)

str(NHANES)

#WTMEC2YR indicated full sample 2 year exam weight, only applicable for NHANES
2011-2012 study

weights<-NHANES$WTMEC2YR

weights<-as.matrix(weights)

#column numbers of covariates and FVC-associated WBC subtypes

x<-NHANES[c(5:11,13:16)]

x<-as.matrix(x)

y<-NHANES$FVC

#covariates were included in the LASSO regression without penalization

p.fac<-rep(1,ncol(x))

p.fac[c(1:6)]<-0

set.seed(91)


fit<-glmnet(x,y)

plot(fit, label=TRUE)

print(fit)

plot(fit,xvar="lambda",label=TRUE)
```

```
cvfit<-
cv.glmnet(x ,y,family="gaussian",penalty.factor=p.fac,type.measure="mse",intercept=TRUE,
nfolds=10)
plot(cvfit)
print(cvfit)
# WBC subtypes selected at minimum mean squared error
cvfit$lambda.min
coef(cvfit,s="lambda.min")
```

3. SAS codes for multiple-marker regression analysis

```
/*NHANES -2012 study*/
PROC SURVEYREG data=NHANES nomcar;
STRATA SDMVSTRA;
CLUSTER SDMVPSU;
WEIGHT WTMEC2YR;
CLASS race;
MODEL FVC=age sex race height smoke drink exercise
WBC_subtypes_selected_by_LASSO/clparm solution vadjust=none;
run;
/*COW and DFTJ studies, analyzing code in the COW study was listed as an example*/
PROC GLM data=COW;
```

```
MODEL FVC =age sex height smoke drink exercise
WBC_subtypes_selected_by_LASSO/clparm solution;
run;
```

4. SAS codes for interaction analysis

```
/*NHANES -2012 study, analyzing code for interaction of total WBC counts with sex on
FVC was listed as an example*/
PROC SURVEYREG data=NHANES nomcar;
STRATA SDMVSTRA;
CLUSTER SDMVPSU;
WEIGHT WTMEC2YR;
CLASS race;
MODEL FVC=age sex race height smoke drink exercise WBC sex*WBC/clparm solution
vadjust=none;
run;
/*COW and DFTJ studies, analyzing code for interaction of total WBC counts with sex on
FVC in the COW study was listed as an example*/
PROC GLM data=COW;
MODEL FVC =age sex height smoke drink exercise WBC sex*WBC/clparm solution;
run;
```

5. R codes for MR analysis

```
library(MendelianRandomization)

mrdata=read.table("WBC_FVC.txt", sep="\t", header=T)

head(mrdata)

dim(mrdata)

MRInputObject<-mr_input(bx=mrdata[,'WBC'], bxse=mrdata[,'WBC_SE'],

by=mrdata[,'FVC'], byse=mrdata[,'FVC_SE'])

MRAll<-mr_allmethods(MRInputObject)

MRAll


#calculate Cochran's Q and heterogeneity P

MBEObject<-mr_ivw(mr_input(bx=mrdata[,'WBC'], bxse=mrdata[,'WBC_SE'],

by=mrdata[,'FVC'], byse=mrdata[,'FVC_SE']))

MBEObject<-mr_egger(mr_input(bx=mrdata[,'WBC'], bxse=mrdata[,'WBC_SE'],

by=mrdata[,'FVC'], byse=mrdata[,'FVC_SE']))


# MR-PRESSO test

MR<-mr_presso(BetaOutcome="FVC", BetaExposure="WBC", SdOutcome="FVC_SE",

SdExposure="WBC_SE", OUTLIERtest=TRUE, DISTORTIONtest=TRUE, data=mrdata,

NbDistribution=5000, SignifThreshold=0.05)

MR$"Main MR results"

MR$"MR-PRESSO results"
```

Table S1 Pearson's correlation coefficients between different WBC subtypes.

	Total WBC	Neutrophils	Lymphocytes	Monocytes	Eosinophils*	Basophils*
**NHANES 2011-2012 (n=3,570)**						
Total WBC	1	0.918	0.550	0.515	0.263	0.291
Neutrophils		1	0.225	0.386	0.141	0.203
Lymphocytes			1	0.341	0.216	0.231
Monocytes				1	0.231	0.147
Eosinophils*					1	0.175
Basophils*						1
**COW (n=1,762)**						
Total WBC	1	0.588	0.892			
Neutrophils		1	0.213			
Lymphocytes			1			
**DFTJ (n=13,827)**						
Total WBC	1	0.886	0.523	0.455	0.310	0.264
Neutrophils		1	0.142	0.327	0.180	0.241
Lymphocytes			1	0.229	0.191	0.044
Monocytes				1	0.212	0.080
Eosinophils*					1	0.193
Basophils*						1

Abbreviations: WBC, white blood cell; NHANES 2011-2012, National Health and Nutrition Examination Survey 2011-2012; COW, coke-oven workers cohort; DFTJ, Dongfeng-Tongji cohort.

Note: All the P <0.001 for the above correlation coefficients.

*Eosinophil and basophil counts were transformed by common logarithm (log10) to approximate normal distribution.

Table S2 Relationships of total and differential WBC counts with lung function among subjects without cancers (single-marker model).

Total and differential WBC counts	FVC, mL		FEV_1, mL	
	β (95% CI)	*P*	β (95% CI)	*P*
**NHANES 2011-2012* (n=3,336)**				
Total WBC	−23.18 (−36.77, −9.58)	0.002	−33.42 (−48.97, −17.87)	<0.001
Neutrophils	−17.24 (−29.89, −4.60)	0.010	−29.43 (−44.80, −14.06)	0.001
Lymphocytes	−58.12 (−109.13, −7.12)	0.028	−63.62 (−113.55, −13.70)	0.016
Monocytes	−329.95 (−529.15, −130.75)	0.003	−372.90 (−568.28, −177.52)	0.001
Eosinophils†	−190.52 (−306.93, −74.11)	0.003	−219.16 (−323.35, −114.96)	<0.001
Basophils†	−19.71 (−124.45, 85.04)	0.696	−48.12 (−159.82, 63.59)	0.376
**COW* (n=1,749)**				
Total WBC	−30.23 (−48.83, −11.64)	0.002	−32.80 (−48.50, −17.11)	<0.001
Neutrophils	−40.99 (−66.00, −15.97)	0.001	−37.13 (−58.28, −15.99)	0.001
Lymphocytes	−37.62 (−86.17, 10.93)	0.129	−55.25 (−96.35, −14.15)	0.008
**DFTJ* (n=13,224)**				
Total WBC	−42.26 (−49.35, −35.17)	<0.001	−23.95 (−30.11, −17.80)	<0.001
Neutrophils	−51.53 (−60.95, −42.12)	<0.001	−28.79 (−36.95, −20.63)	<0.001
Lymphocytes	−38.91 (−57.55, −20.26)	<0.001	−19.54 (−35.65, −3.43)	0.018
Monocytes	−237.83 (−317.54, −158.13)	<0.001	−178.93 (−247.78, −110.08)	<0.001
Eosinophils†	−88.45 (−118.34, −58.56)	<0.001	−87.33 (−113.14, −61.53)	<0.001
Basophils†	−176.89 (−205.78, −147.99)	<0.001	−58.00 (−83.10, −32.89)	<0.001

Abbreviations: WBC, white blood cell; FVC, forced vital capacity; FEV_1, forced expiratory volume in one second; NHANES 2011-2012, National Health and Nutrition Examination Survey 2011-2012; DFTJ, Dongfeng-Tongji cohort.

Notes: *Total and differential WBC counts were included in the multiple linear regression model separately, and the model was adjusted for age, sex, race (only in NHANES 2011-2012 population), height, smoking, alcohol use, and exercise.

†Eosinophil and basophil counts were transformed by common logarithm (log10) to approximate normal distribution.

Table S3 Relationships of total and differential WBC counts with lung function among subjects without using anti-infectious drugs (single-marker model).

Total and differential WBC counts	FVC, mL		FEV_1, mL	
	β (95% CI)	*P*	β (95% CI)	*P*
**NHANES 2011-2012* (n=3,403)**				
Total WBC	−23.25 (−36.08, −10.42)	0.001	−34.10 (−50.92, −17.28)	0.001
Neutrophils	−18.40 (−31.66, −5.13)	0.009	−30.73 (−47.44, −14.02)	0.001
Lymphocytes	−53.21 (−98.16, −8.25)	0.023	−64.10 (−114.45, −13.75)	0.016
Monocytes	−343.47 (−505.78, −181.16)	<0.001	−391.69 (−578.76, −204.63)	<0.001
Eosinophils†	−184.36 (−286.77, −81.94)	0.001	−221.94 (−321.42, −122.47)	<0.001
Basophils†	2.31 (−101.86, 106.47)	0.963	−26.99 (−139.07, 85.10)	0.618
**DFTJ* (n=12,598)**				
Total WBC	−42.16 (−49.42, −34.89)	<0.001	−24.20 (−30.46, −17.95)	<0.001
Neutrophils	−51.79 (−61.42, −42.15)	<0.001	−29.66 (−37.95, −21.36)	<0.001
Lymphocytes	−39.55 (−58.67, −20.44)	<0.001	−20.69 (−37.10, −4.28)	0.014
Monocytes	−243.63 (−325.43, −161.83)	<0.001	−181.44 (−251.65, −111.23)	<0.001
Eosinophils†	−75.52 (−106.18, −44.86)	<0.001	−72.67 (−98.97, −46.37)	<0.001
Basophils†	−177.26 (−206.85, −147.67)	<0.001	−56.35 (−81.90, −30.80)	<0.001

Abbreviations: WBC, white blood cell; FVC, forced vital capacity; FEV_1, forced expiratory volume in one second; NHANES 2011-2012, National Health and Nutrition Examination Survey 2011-2012; DFTJ, Dongfeng-Tongji cohort.

Notes: *Total and differential WBC counts were included in the multiple linear regression model separately, and the model was adjusted for age, sex, race (only in NHANES 2011-2012 population), height, smoking, alcohol use, and exercise.

†Eosinophil and basophil counts were transformed by common logarithm (log10) to approximate normal distribution.

Table S4 Characteristics of SNPs associated with total and differential WBC counts in published GWAS.

WBC and subtypes	SNPs	Chr:Position	Gene	Ref/Alt	Alt frequency	β (SE)	*P*	F statistics
Total WBC								
	rs3917932	1:36943916	*CSF3R*	C/G	0.576	−0.037 (0.004)	5.09×10^{-17}	70.30
	rs1875763	1:161696998	*FCRLB*	G/C	0.686	−0.027 (0.005)	1.90×10^{-8}	31.59
	rs1260326	2:27730940	*GCKR*	T/C	0.441	−0.026 (0.004)	2.01×10^{-9}	35.960
	rs75874749	2:136713108	*DARS*	C/T	0.848	−0.067 (0.006)	6.75×10^{-30}	129.010
	rs12052715	2:160677375	*LY75, LY75-CD302*	C/G	0.919	−0.048 (0.008)	5.46×10^{-10}	38.51
	rs6740847	2:182308352	*LOC101927156, ITGA4*	G/A	0.665	−0.025 (0.004)	2.61×10^{-8}	30.990
	rs78107966	2:219062001	*CXCR1, ARPC2*	T/C	0.191	−0.038 (0.006)	2.54×10^{-11}	44.51
	rs7628747	3:47001990	*CCDC12*	T/A	0.453	−0.026 (0.004)	1.08×10^{-9}	37.19
	rs10016631	4:57885963	*POLR2B*	A/G	0.501	−0.024 (0.004)	9.94×10^{-9}	32.86
	rs549280	4:74971196	*CXCL2, MTHFD2L*	G/A	0.563	−0.044 (0.004)	9.23×10^{-25}	105.52
	rs6832779	4:83556631	*SCD5*	A/G	0.313	−0.036 (0.005)	1.22×10^{-13}	54.97
	rs4449583	5:1284135	*TERT*	T/C	0.653	−0.031 (0.005)	2.39×10^{-10}	40.13
	rs1322599	6:16758425	*ATXN1*	C/T	0.620	−0.028 (0.005)	1.53×10^{-9}	36.50
	rs114398276*	6:31246823	*HLA-C, HLA-B*	T/A	0.872	−0.067 (0.006)	9.98×10^{-25}	105.41
	rs9368924	6:36353934	*ETV7*	A/G	0.370	−0.027 (0.004)	5.61×10^{-10}	38.43
	rs9399136	6:135402339	*HBS1L, MYB*	T/C	0.343	−0.042 (0.004)	4.23×10^{-21}	88.890
	rs57123607	7:28717030	*CREB5*	T/C	0.732	−0.041 (0.005)	1.84×10^{-17}	72.32
	rs445	7:92408370	*CDK6*	C/T	0.312	−0.081 (0.005)	1.58×10^{-70}	315.200
	rs11776339	8:56800362	*LYN*	C/T	0.622	−0.027 (0.005)	3.73×10^{-8}	30.28
	rs13280978	8:61704055	*CHD7*	C/T	0.771	−0.037 (0.005)	2.76×10^{-13}	53.370
	rs1433578	8:130601089	*CCDC26*	T/C	0.174	−0.057 (0.006)	9.37×10^{-25}	105.54
	rs17694933	9:22164309	*CDKN2B-AS1, DMRTA1*	A/G	0.625	−0.031 (0.004)	2.40×10^{-12}	49.12
	rs60980157	9:139235415	*GPSM1*	T/C	0.878	−0.041 (0.007)	2.96×10^{-8}	30.73

Table S4 Characteristics of SNPs associated with total and differential WBC counts in published GWAS.

WBC and subtypes	SNPs	Chr:Position	Gene	Ref/Alt	Alt frequency	β (SE)	*P*	F statistics
	rs3747869	10:73520632	*C10orf54*	C/A	0.156	−0.035 (0.006)	2.12×10^{-9}	35.860
	rs12357266	10:98978045	*ARHGAP19-SLIT1*	A/G	0.351	−0.027 (0.004)	1.33×10^{-9}	36.79
	rs35879747	10:104348694	*SUFU*	A/T	0.905	−0.044 (0.007)	4.07×10^{-9}	34.60
	rs77652395	10:111947275	*ADD3, MXI1*	T/G	0.649	−0.028 (0.005)	7.14×10^{-10}	37.99
	rs4237543*	11:47454152	*PSMC3, RAPSN*	T/A	0.515	−0.027 (0.005)	8.72×10^{-9}	33.11
	rs75963851	11:95096321	*LOC100129203, FAM76B*	A/G	0.073	−0.061 (0.008)	2.24×10^{-13}	53.78
	rs2040571	12:111357727	*MYL2*	A/G	0.779	−0.040 (0.006)	1.15×10^{-12}	50.56
	rs8017228	14:25449226	*STXBP6*	C/A	0.522	−0.033 (0.004)	3.97×10^{-15}	61.710
	rs3915470	15:80279344	*BCL2A1, ZFAND6*	A/G	0.708	−0.040 (0.005)	3.02×10^{-16}	66.79
	rs55771023	17:38165298	*PSMD3, CSF3*	A/C	0.484	−0.070 (0.004)	9.43×10^{-62}	274.97
	rs17841683	18:42084513	*LINC01478*	G/C	0.748	−0.031 (0.005)	7.75×10^{-10}	37.83
	rs55688536	19:1123176	*SBNO2*	G/A	0.132	−0.037 (0.006)	1.29×10^{-8}	32.35
	rs311629	19:3180803	*S1PR4*	G/A	0.269	−0.040 (0.006)	2.25×10^{-13}	53.77
Neutrophils								
	rs2994007	1:36880829	*LSM10, OSCP1*	G/A	0.608	−0.047 (0.006)	4.37×10^{-16}	66.07
	rs149881318	2:136695772	*DARS*	C/T	0.851	−0.087 (0.008)	6.71×10^{-28}	119.87
	rs12469306	2:160705485	*LY75, LY75-CD302*	A/G	0.825	−0.046 (0.008)	2.51×10^{-9}	35.53
	rs78107966	2:219062001	*CXCR1, ARPC2*	T/C	0.191	−0.055 (0.008)	3.73×10^{-13}	52.78
	rs549280	4:74971196	*CXCL2, MTHFD2L*	G/A	0.563	−0.063 (0.006)	2.33×10^{-28}	121.98
	rs4449583	5:1284135	*TERT*	T/C	0.653	−0.042 (0.007)	1.78×10^{-10}	40.69
	rs562760	6:16750011	*ATXN1*	G/T	0.671	−0.035 (0.006)	6.34×10^{-9}	33.74
	rs115681968	6:31272553	*HLA-C, HLA-B*	A/G	0.872	−0.074 (0.009)	2.55×10^{-17}	71.67
	rs886816	7:28723407	*CREB5*	A/G	0.784	−0.066 (0.007)	7.72×10^{-20}	83.11
	rs445	7:92408370	*CDK6*	C/T	0.312	−0.087 (0.006)	3.84×10^{-46}	203.36

Table S4 Characteristics of SNPs associated with total and differential WBC counts in published GWAS.

WBC and subtypes	SNPs	Chr:Position	Gene	Ref/Alt	Alt frequency	β (SE)	*P*	F statistics
	rs4237036	8:61701057	*CHD7*	C/T	0.771	−0.051 (0.007)	4.05×10^{-14}	57.16
	rs75418630	8:130557032	*CCDC26*	A/G	0.123	−0.074 (0.009)	1.69×10^{-17}	72.47
	rs12357266	10:98978045	*ARHGAP19-SLIT1*	A/G	0.351	−0.037 (0.006)	7.24×10^{-10}	37.95
	rs77652395	10:111947275	*ADD3, MXI1*	T/G	0.649	−0.033 (0.006)	4.38×10^{-8}	29.98
	rs79208462	12:110098272	*MVK, FAM222A*	G/A	0.930	−0.070 (0.012)	6.23×10^{-9}	33.74
	rs2040571	12:111357727	*MYL2*	A/G	0.779	−0.062 (0.008)	2.92×10^{-16}	66.87
	rs7141943	14:25438250	*STXBP6*	G/A	0.511	−0.032 (0.006)	1.15×10^{-8}	32.58
	rs709591	17:38175561	*MED24*	A/T	0.587	−0.091 (0.006)	3.67×10^{-56}	249.29
	rs12606438	18:42041063	*LINC01478*	C/A	0.740	−0.042 (0.006)	1.39×10^{-10}	41.19
	rs2301813	19:1000799	*GRIN3B*	C/G	0.245	−0.039 (0.007)	2.68×10^{-8}	30.93
	rs368975	19:3216946	*NCLN, CELF5*	G/A	0.130	−0.061 (0.009)	9.01×10^{-12}	46.54
Monocytes								
	rs4970966	1:150584103	*MCL1, ENSA*	G/T	0.181	−0.047 (0.007)	1.43×10^{-10}	41.12
	rs3789087	2:111791653	*ACOXL*	C/T	0.178	−0.048 (0.008)	1.27×10^{-10}	41.35
	rs6711493	2:136692553	*DARS*	G/A	0.691	−0.038 (0.006)	8.82×10^{-10}	37.57
	rs1449263	2:182319301	*LOC101927156, ITGA4*	T/C	0.627	−0.098 (0.006)	8.40×10^{-63}	279.76
	rs2228468*	3:42907112	*ACKR2*	A/C	0.597	−0.044 (0.006)	2.36×10^{-14}	58.20
	rs62242983	3:46383922	*CCR3, CCR2*	T/C	0.259	−0.054 (0.007)	7.25×10^{-14}	56.01
	rs2734037	3:128308884	*LINC01565, RPN1*	T/C	0.579	−0.052 (0.006)	4.37×10^{-19}	79.69
	rs6815294	4:7042349	*LOC100129931*	A/G	0.537	−0.040 (0.006)	9.28×10^{-12}	46.48
	rs115007843	4:83575212	*SCD5*	A/C	0.342	−0.053 (0.006)	3.48×10^{-17}	71.05
	rs114208039	6:31241639	*HLA-C, HLA-B*	A/G	0.577	−0.048 (0.006)	3.35×10^{-17}	71.11
	rs73057397	7:6504240	*KDELR2*	C/T	0.615	−0.039 (0.006)	8.67×10^{-11}	42.09
	rs10276619	7:50313352	*C7orf72, IKZF1*	G/A	0.554	−0.037 (0.006)	1.23×10^{-10}	41.42

Table S4 Characteristics of SNPs associated with total and differential WBC counts in published GWAS.

WBC and subtypes	SNPs	Chr:Position	Gene	Ref/Alt	Alt frequency	β (SE)	*P*	F statistics
	rs445	7:92408370	*CDK6*	C/T	0.312	−0.053 (0.006)	6.91×10^{-18}	74.23
	rs35389394	8:130621254	*CCDC26*	C/T	0.547	−0.077 (0.006)	8.21×10^{-41}	178.91
	rs7041895	9:22162794	*CDKN2B-AS1, DMRTA1*	C/A	0.616	−0.048 (0.006)	4.30×10^{-16}	66.08
	rs10980797	9:113912553	*LPAR1, MIR7702*	A/G	0.145	−0.065 (0.008)	9.03×10^{-16}	64.63
	rs10987830	9:130778738	*FAM102A, NAIF1*	A/G	0.857	−0.054 (0.009)	1.91×10^{-10}	40.56
	rs687621	9:136137065	*ABO*	A/G	0.463	−0.032 (0.006)	2.23×10^{-8}	31.28
	rs6584283	10:101290301	*LINC01475*	T/C	0.592	−0.035 (0.006)	1.26×10^{-9}	36.87
	rs75963851	11:95363157	*LOC100129203, FAM76B*	A/G	0.073	−0.070 (0.011)	3.59×10^{-10}	39.29
	rs9551434	13:28641571	*FLT3*	A/G	0.183	−0.060 (0.008)	3.80×10^{-15}	61.79
	rs1892548	13:41002641	*LINC00598*	T/C	0.767	−0.039 (0.007)	1.94×10^{-8}	31.56
	rs2239630	14:23589349	*CEBPE*	G/A	0.503	−0.037 (0.006)	4.44×10^{-10}	38.910
	rs2004925	14:25445362	*STXBP6*	A/G	0.514	−0.047 (0.006)	1.64×10^{-16}	68.00
	rs12898000	14:103835128	*EIF5, MARK3*	C/G	0.637	−0.043 (0.006)	3.74×10^{-12}	48.240
	rs57556178	15:42242888	*EHD4*	G/A	0.147	−0.045 (0.008)	3.82×10^{-8}	30.25
	rs117424492	15:80267395	*BCL2A1, ZFAND6*	C/G	0.702	−0.034 (0.006)	3.44×10^{-8}	30.440
	rs9674233	16:86012599	*IRF8, LINC01082*	T/C	0.519	−0.053 (0.006)	1.66×10^{-20}	86.16
	rs58681483	17:57934654	*MIR21, TUBD1*	A/G	0.122	−0.062 (0.009)	1.28×10^{-12}	50.35
	rs12461821	19:6671369	*TNFSF14*	G/A	0.386	−0.038 (0.006)	6.56×10^{-11}	42.65
	rs4811020	20:48903034	*LINC01272, LINC01270*	C/T	0.115	−0.055 (0.009)	7.32×10^{-10}	37.93
Eosinophils								
	rs8191981	1:154941263	*SHC1*	G/A	0.042	−0.188 (0.015)	3.75×10^{-37}	162.14
	rs6684992	1:87752000	*LOC101927844, LMO4*	T/A	0.663	−0.037 (0.006)	6.55×10^{-10}	38.15
	rs6543119	2:102963072	*IL1RL1*	T/A	0.590	−0.060 (0.006)	6.03×10^{-25}	106.40
	rs13022407	2:213836206	*MIR4776-2, IKZF2*	T/C	0.685	−0.055 (0.006)	6.20×10^{-19}	79.00

Table S4 Characteristics of SNPs associated with total and differential WBC counts in published GWAS.

WBC and subtypes	SNPs	Chr:Position	Gene	Ref/Alt	Alt frequency	β (SE)	*P*	F statistics
	rs35480293	3:33087395	*GLB1*	A/C	0.466	−0.040 (0.006)	3.75×10^{-12}	48.27
	rs7646596	3:128320345	*LINC01565, RPN1*	G/T	0.355	−0.096 (0.006)	3.17×10^{-56}	249.63
	rs2078387	5:131884894	*IL5, RAD50*	C/A	0.783	−0.062 (0.007)	8.88×10^{-19}	78.29
	rs114965247	6:31350303	*HLA-B, MICA*	T/G	0.221	−0.072 (0.007)	5.35×10^{-25}	106.63
	rs2210366	6:135415208	*HBS1L, MYB*	A/G	0.637	−0.058 (0.006)	1.55×10^{-22}	95.40
	rs9986877	7:20544739	*ITGB8, ABCB5*	T/A	0.242	−0.059 (0.007)	3.10×10^{-18}	75.82
	rs445	7:92408370	*CDK6*	C/T	0.312	−0.051 (0.006)	5.22×10^{-17}	70.23
	rs6994642	8:61723777	*CHD7*	C/T	0.183	−0.042 (0.008)	3.16×10^{-8}	30.61
	rs4562281	8:130651646	*CCDC26*	G/C	0.681	−0.038 (0.006)	4.11×10^{-10}	39.06
	rs16917546	10:64397538	*ZNF365*	T/C	0.502	−0.036 (0.006)	3.92×10^{-10}	39.15
	rs7114362	11:76293070	*EMSY, LRRC32*	T/C	0.634	−0.034 (0.006)	9.56×10^{-9}	32.92
	rs117072053	15:80267481	*BCL2A1, ZFAND6*	T/C	0.702	−0.040 (0.006)	1.54×10^{-10}	40.98
	rs9901392	17:4604836	*PELP1*	C/T	0.874	−0.047 (0.009)	3.52×10^{-8}	30.39
	rs7249415	19:40223579	*CLC*	A/C	0.612	−0.043 (0.006)	1.16×10^{-12}	50.540
Basophils								
	rs6703781	1:87748885	*LOC101927844, LMO4*	G/A	0.335	−0.061 (0.006)	1.30×10^{-24}	104.89
	rs6427756	1:199000727	*LINC01221*	A/G	0.823	−0.041 (0.007)	2.40×10^{-8}	31.14
	rs4951254	1:205642390	*SLC45A3*	C/T	0.447	−0.046 (0.006)	3.36×10^{-15}	62.04
	rs16823866	2:145324977	*LINC01412*	T/C	0.270	−0.056 (0.006)	4.20×10^{-18}	75.22
	rs3770136	2:182329460	*ITGA4*	C/T	0.228	−0.037 (0.007)	4.10×10^{-8}	30.11
	rs3804785	3:3205142	*CRBN*	G/T	0.263	−0.062 (0.007)	2.84×10^{-20}	85.08
	rs6782812	3:128317997	*LINC01565, RPN1*	A/G	0.346	−0.142 (0.006)	2.10×10^{-123}	558.29
	rs73069193	3:194138782	*ATP13A3*	A/G	0.062	−0.067 (0.012)	4.21×10^{-8}	30.06
	rs1408	4:88057353	*AFF1*	G/A	0.556	−0.032 (0.006)	1.37×10^{-8}	32.23

Table S4 Characteristics of SNPs associated with total and differential WBC counts in published GWAS.

WBC and subtypes	SNPs	Chr:Position	Gene	Ref/Alt	Alt frequency	β (SE)	*P*	F statistics
	rs113721499	6:31229307	*HCG27, HLA-C*	C/T	0.764	−0.07 (0.008)	2.00×10^{-20}	85.77
	rs12208785	6:109703027	*CD164*	G/A	0.779	−0.043 (0.007)	4.14×10^{-10}	39.05
	rs7008536	8:119113134	*EXT1*	C/A	0.564	−0.047 (0.006)	4.12×10^{-16}	66.17
	rs2980884	8:126474356	*TRIB1, LINC00861*	A/G	0.746	−0.037 (0.007)	1.56×10^{-8}	31.98
	rs10100356	8:130626164	*CCDC26*	A/G	0.543	−0.034 (0.006)	5.36×10^{-9}	34.06
	rs741804	10:89656156	*PTEN*	A/C	0.193	−0.048 (0.008)	1.83×10^{-10}	40.65
	rs150876292	11:69689681	*FGF3, LOC101928443*	T/C	0.098	−0.062 (0.011)	3.73×10^{-8}	30.27
	rs11018874	11:89875437	*NAALAD2*	G/A	0.266	−0.055 (0.007)	2.27×10^{-16}	67.35
	rs695113	11:128562098	*SENCR*	T/C	0.342	−0.044 (0.006)	2.09×10^{-13}	53.92
	rs7315361	12:66671779	*IRAK3, HELB*	A/T	0.53	−0.036 (0.006)	2.21×10^{-10}	40.27
	rs61123801	13:41390781	*TPTE2P5*	G/A	0.211	−0.041 (0.007)	1.30×10^{-8}	32.34
	rs1474920	14:23587555	*CEBPE*	T/G	0.713	−0.059 (0.006)	6.08×10^{-21}	88.12
	rs11857230	15:50392821	*ATP8B4*	A/T	0.312	−0.044 (0.006)	5.51×10^{-13}	52.01
	rs12445547	16:88518569	*ZNF469, ZFPM1*	T/G	0.497	−0.043 (0.006)	5.93×10^{-14}	56.40
	rs7253959	19:33755084	*SLC7A10, CEBPA*	A/G	0.474	−0.046 (0.006)	3.24×10^{-13}	53.07
	rs28530618	20:31275581	*C20orf203, COMMD7*	G/A	0.767	−0.038 (0.007)	4.19×10^{-8}	30.06
	rs78762153	21:39845275	*ERG*	C/T	0.922	−0.152 (0.011)	1.06×10^{-43}	192.43

Abbreviation: WBC, white blood cell; GWAS, genome-wide association study.

Notes: Summary statistics were derived from the largest GWAS of total and differential WBC in the Asian population [5].
Total WBC counts were standardized by Z-score, and the study sample size was 107,964.
Neutrophil, monocyte, eosinophil, and basophil counts were standardized by rank-based inverse normal transformation and the study sample size for the genome-wide analysis was 62,076.

*These SNPs were not genotyped or imputed in the participants of present research.

Table S5 The associations of total and differential WBC counts related SNPs with confounders in the COW and DFTJ studies.

WBC and subtypes	SNPs	Ref/Alt	Height		Smoking		Alcohol drinking		Exercise	
			β (95% CI)	*P*	β (95% CI)	*P*	β (95% CI)	*P*	β (95% CI)	*P*
Total WBC										
	rs3917932	C/G	−0.08 (−0.32, 0.16)	0.514	−0.01 (−0.12, 0.11)	0.875	−0.06 (−0.16, 0.04)	0.243	0.06 (−0.07, 0.18)	0.381
	rs1875763	G/C	−0.07 (−0.34, 0.20)	0.628	0.11 (−0.02, 0.24)	0.109	0.07 (−0.05, 0.18)	0.249	0.11 (−0.03, 0.25)	0.130
	rs1260326	T/C	0.05 (−0.19, 0.28)	0.680	−0.02 (−0.13, 0.1)	0.769	0.10 (0, 0.20)	0.044	0.10 (−0.03, 0.22)	0.128
	rs75874749	C/T	0.13 (−0.16, 0.42)	0.371	0.02 (−0.12, 0.15)	0.801	−0.10 (−0.22, 0.02)	0.112	−0.06 (−0.21, 0.09)	0.439
	rs12052715	C/G	−0.28 (−0.64, 0.08)	0.125	0.11 (−0.06, 0.28)	0.197	0.07 (−0.09, 0.22)	0.408	−0.04 (−0.23, 0.15)	0.651
	rs6740847	G/A	−0.08 (−0.33, 0.18)	0.541	0 (−0.12, 0.12)	0.976	−0.07 (−0.18, 0.03)	0.182	−0.04 (−0.18, 0.09)	0.530
	rs78107966	T/C	−0.20 (−0.48, 0.07)	0.148	0.05 (−0.08, 0.19)	0.429	−0.02 (−0.14, 0.10)	0.754	−0.14 (−0.28, 0.01)	0.058
	rs7628747	T/A	−0.01 (−0.25, 0.22)	0.917	0.03 (−0.08, 0.15)	0.581	0.04 (−0.07, 0.14)	0.479	0.10 (−0.02, 0.22)	0.110
	rs10016631	A/G	0.04 (−0.19, 0.27)	0.737	−0.05 (−0.16, 0.07)	0.424	0 (−0.10, 0.10)	0.989	−0.04 (−0.16, 0.08)	0.546
	rs549280	G/A	0.03 (−0.20, 0.27)	0.798	0.03 (−0.08, 0.14)	0.602	−0.06 (−0.16, 0.05)	0.282	−0.01 (−0.14, 0.11)	0.824
	rs6832779	A/G	−0.13 (−0.38, 0.11)	0.283	−0.11 (−0.23, 0.01)	0.074	0.05 (−0.05, 0.16)	0.323	−0.03 (−0.15, 0.10)	0.701
	rs4449583	T/C	0 (−0.24, 0.24)	0.983	0.06 (−0.06, 0.17)	0.338	0.05 (−0.06, 0.15)	0.361	0.08 (−0.05, 0.20)	0.234
	rs1322599	C/T	−0.01 (−0.26, 0.25)	0.949	−0.05 (−0.17, 0.07)	0.408	−0.01 (−0.12, 0.09)	0.798	0.04 (−0.09, 0.17)	0.521
	rs9368924	A/G	−0.10 (−0.34, 0.14)	0.425	0.05 (−0.07, 0.16)	0.422	−0.06 (−0.16, 0.05)	0.281	−0.02 (−0.15, 0.11)	0.750
	rs9399136	T/C	0.32 (0.07, 0.58)	0.014	0.07 (−0.05, 0.20)	0.248	0.05 (−0.06, 0.16)	0.332	−0.01 (−0.15, 0.12)	0.846
	rs57123607	T/C	0.02 (−0.27, 0.31)	0.883	−0.05 (−0.19, 0.09)	0.509	0.14 (0.01, 0.27)	0.030	−0.06 (−0.22, 0.09)	0.426
	rs445	C/T	−0.11 (−0.35, 0.14)	0.390	0.11 (−0.01, 0.23)	0.064	0.09 (−0.02, 0.19)	0.109	−0.02 (−0.15, 0.11)	0.751
	rs11776339	C/T	0.07 (−0.19, 0.33)	0.621	0.02 (−0.11, 0.14)	0.773	−0.05 (−0.17, 0.06)	0.340	−0.02 (−0.16, 0.11)	0.745
	rs13280978	C/T	−0.04 (−0.34, 0.25)	0.770	0.15 (0.01, 0.29)	0.036	0 (−0.13, 0.12)	0.954	−0.05 (−0.20, 0.10)	0.517
	rs1433578	T/C	−0.32 (−0.67, 0.03)	0.073	0.04 (−0.13, 0.2)	0.686	−0.05 (−0.20, 0.10)	0.490	0.01 (−0.17, 0.19)	0.898
	rs17694933	A/G	0.02 (−0.23, 0.27)	0.858	0.09 (−0.03, 0.21)	0.155	0.01 (−0.10, 0.12)	0.843	−0.09 (−0.22, 0.05)	0.197
	rs60980157	T/C	0.43 (−0.28, 1.15)	0.235	−0.24 (−0.6, 0.12)	0.197	−0.26 (−0.56, 0.05)	0.104	−0.05 (−0.44, 0.33)	0.787
	rs3747869	C/A	0.11 (−0.18, 0.41)	0.451	0.07 (−0.07, 0.21)	0.327	−0.04 (−0.16, 0.09)	0.546	0.04 (−0.11, 0.19)	0.608

Table S5 The associations of total and differential WBC counts related SNPs with confounders in the COW and DFTJ studies.

WBC and subtypes	SNPs	Ref/Alt	Height		Smoking		Alcohol drinking		Exercise	
			β (95% CI)	*P*	β (95% CI)	*P*	β (95% CI)	*P*	β (95% CI)	*P*
	rs12357266	A/G	0.16 (−0.11, 0.42)	0.247	−0.02 (−0.14, 0.11)	0.816	−0.01 (−0.12, 0.11)	0.910	0.04 (−0.10, 0.18)	0.571
	rs35879747	A/T	−0.01 (−0.34, 0.32)	0.947	0.15 (−0.01, 0.3)	0.061	0.04 (−0.10, 0.18)	0.575	−0.05 (−0.23, 0.12)	0.554
	rs77652395	T/G	0.31 (0.06, 0.56)	0.016	−0.01 (−0.13, 0.11)	0.890	0.07 (−0.04, 0.18)	0.208	0.02 (−0.11, 0.15)	0.795
	rs75963851	A/G	0.66 (0.10, 1.22)	0.022	−0.1 (−0.37, 0.18)	0.499	−0.03 (−0.27, 0.22)	0.839	0 (−0.30, 0.31)	0.978
	rs2040571*	A/G	0.19 (−0.11, 0.49)	0.208	0.15 (0.01, 0.29)	0.042	**0.77 (0.63, 0.91)**	**<0.001**	−0.10 (−0.26, 0.06)	0.216
	rs8017228	C/A	−0.10 (−0.34, 0.13)	0.388	−0.05 (−0.17, 0.06)	0.362	−0.01 (−0.11, 0.09)	0.803	0.03 (−0.09, 0.15)	0.602
	rs3915470	A/G	−0.13 (−0.39, 0.13)	0.319	0.05 (−0.08, 0.17)	0.474	−0.10 (−0.21, 0.01)	0.084	−0.04 (−0.17, 0.10)	0.577
	rs55771023	A/C	0.04 (−0.20, 0.28)	0.764	−0.02 (−0.14, 0.09)	0.680	0.07 (−0.03, 0.17)	0.190	0.01 (−0.11, 0.14)	0.867
	rs17841683	G/C	0.23 (−0.07, 0.53)	0.137	0.06 (−0.09, 0.20)	0.419	0.01 (−0.12, 0.13)	0.929	−0.02 (−0.18, 0.14)	0.788
	rs55688536	G/A	0.07 (−0.29, 0.42)	0.714	0.02 (−0.15, 0.19)	0.802	0.15 (0, 0.30)	0.050	0.14 (−0.05, 0.32)	0.161
	rs311629	G/A	0.11 (−0.18, 0.40)	0.443	0.02 (−0.12, 0.16)	0.766	0.07 (−0.05, 0.19)	0.269	0.09 (−0.06, 0.24)	0.224
Neutrophils										
	rs2994007	G/A	−0.04 (−0.28, 0.21)	0.778	−0.08 (−0.20, 0.04)	0.181	−0.03 (−0.14, 0.07)	0.525	0.07 (−0.05, 0.20)	0.253
	rs149881318	C/T	0.12 (−0.17, 0.40)	0.419	0.01 (−0.12, 0.15)	0.852	−0.10 (−0.22, 0.02)	0.115	−0.07 (−0.22, 0.08)	0.357
	rs12469306	A/G	0 (−0.27, 0.28)	0.979	−0.02 (−0.15, 0.11)	0.743	−0.02 (−0.14, 0.10)	0.736	0.02 (−0.12, 0.17)	0.741
	rs78107966	T/C	−0.21 (−0.49, 0.07)	0.142	0.06 (−0.08, 0.19)	0.416	−0.02 (−0.14, 0.10)	0.760	−0.13 (−0.28, 0.01)	0.063
	rs549280	G/A	0.03 (−0.20, 0.27)	0.797	0.03 (−0.08, 0.14)	0.597	−0.06 (−0.16, 0.05)	0.282	−0.01 (−0.14, 0.11)	0.821
	rs4449583	T/C	0.01 (−0.24, 0.25)	0.969	0.05 (−0.06, 0.17)	0.362	0.05 (−0.06, 0.15)	0.373	0.07 (−0.06, 0.20)	0.267
	rs562760	G/T	−0.02 (−0.29, 0.26)	0.907	0 (−0.13, 0.13)	0.949	0.01 (−0.11, 0.13)	0.857	0.05 (−0.09, 0.19)	0.506
	rs115681968	A/G	0.18 (−0.20, 0.55)	0.357	0.12 (−0.06, 0.31)	0.185	−0.09 (−0.25, 0.07)	0.293	−0.11 (−0.34, 0.13)	0.377
	rs886816	A/G	−0.07 (−0.39, 0.25)	0.681	0 (−0.15, 0.16)	0.976	0.12 (−0.02, 0.26)	0.089	−0.04 (−0.21, 0.13)	0.648
	rs445	C/T	−0.11 (−0.36, 0.14)	0.375	0.11 (−0.01, 0.23)	0.061	0.09 (−0.02, 0.19)	0.107	−0.02 (−0.15, 0.11)	0.777
	rs4237036	C/T	−0.06 (−0.35, 0.23)	0.688	0.15 (0.01, 0.29)	0.039	−0.01 (−0.13, 0.12)	0.901	−0.06 (−0.21, 0.10)	0.462
	rs75418630	A/G	−0.15 (−0.65, 0.34)	0.538	0.06 (−0.18, 0.30)	0.648	0.12 (−0.09, 0.33)	0.259	−0.03 (−0.28, 0.23)	0.830

Table S5 The associations of total and differential WBC counts related SNPs with confounders in the COW and DFTJ studies.

WBC and subtypes	SNPs	Ref/Alt	Height		Smoking		Alcohol drinking		Exercise	
			β (95% CI)	*P*	β (95% CI)	*P*	β (95% CI)	*P*	β (95% CI)	*P*
	rs12357266	A/G	0.15 (−0.11, 0.42)	0.257	−0.01 (−0.14, 0.11)	0.832	−0.01 (−0.12, 0.11)	0.916	0.04 (−0.10, 0.18)	0.549
	rs77652395	T/G	0.32 (0.07, 0.57)	0.014	−0.01 (−0.13, 0.11)	0.847	0.07 (−0.04, 0.18)	0.217	0.01 (−0.12, 0.14)	0.861
	rs79208462*	G/A	0.08 (−0.47, 0.63)	0.778	0.21 (−0.04, 0.47)	0.103	**0.56 (0.31, 0.81)**	**<0.001**	0.14 (−0.14, 0.41)	0.334
	rs2040571*	A/G	0.19 (−0.11, 0.49)	0.210	0.15 (0.01, 0.29)	0.041	**0.77 (0.63, 0.91)**	**<0.001**	−0.10 (−0.26, 0.06)	0.218
	rs7141943	G/A	−0.10 (−0.34, 0.13)	0.391	−0.06 (−0.17, 0.06)	0.334	−0.01 (−0.12, 0.09)	0.780	0.03 (−0.09, 0.16)	0.583
	rs709591	A/T	0.03 (−0.21, 0.27)	0.799	−0.02 (−0.14, 0.09)	0.723	0.07 (−0.04, 0.17)	0.202	0.02 (−0.11, 0.14)	0.793
	rs12606438	C/A	0.21 (−0.08, 0.50)	0.163	0.07 (−0.07, 0.21)	0.327	−0.03 (−0.15, 0.10)	0.678	0.01 (−0.15, 0.16)	0.951
	rs2301813	C/G	0.07 (−0.19, 0.33)	0.588	−0.07 (−0.20, 0.05)	0.247	0.02 (−0.09, 0.13)	0.721	0.04 (−0.10, 0.17)	0.592
	rs368975	G/A	0.29 (−0.10, 0.68)	0.144	−0.12 (−0.30, 0.07)	0.214	−0.06 (−0.23, 0.11)	0.488	−0.21 (−0.41, −0.02)	0.034
Monocytes										
	rs4970966	G/T	0.03 (−0.25, 0.31)	0.829	0.07 (−0.07, 0.20)	0.311	−0.03 (−0.15, 0.09)	0.611	0.09 (−0.06, 0.24)	0.225
	rs3789087	C/T	0.10 (−0.19, 0.38)	0.496	0.08 (−0.06, 0.22)	0.246	0.09 (−0.03, 0.22)	0.123	−0.02 (−0.17, 0.12)	0.743
	rs6711493	G/A	0.14 (−0.10, 0.38)	0.249	0.11 (−0.01, 0.22)	0.071	0.04 (−0.06, 0.14)	0.464	−0.07 (−0.20, 0.05)	0.244
	rs1449263	T/C	−0.05 (−0.31, 0.20)	0.671	−0.03 (−0.15, 0.09)	0.616	−0.05 (−0.16, 0.06)	0.337	−0.01 (−0.14, 0.13)	0.941
	rs62242983	T/C	−0.04 (−0.32, 0.25)	0.791	0.01 (−0.13, 0.15)	0.882	0.03 (−0.09, 0.15)	0.599	−0.10 (−0.24, 0.05)	0.194
	rs2734037	T/C	0.10 (−0.15, 0.36)	0.423	0.03 (−0.09, 0.15)	0.597	0.04 (−0.07, 0.14)	0.510	−0.02 (−0.15, 0.12)	0.812
	rs6815294	A/G	−0.06 (−0.29, 0.18)	0.650	0.07 (−0.05, 0.18)	0.236	−0.06 (−0.16, 0.04)	0.236	0.03 (−0.09, 0.16)	0.607
	rs115007843	A/C	−0.13 (−0.37, 0.11)	0.289	−0.11 (−0.23, 0)	0.058	0.09 (−0.01, 0.20)	0.076	−0.06 (−0.19, 0.06)	0.310
	rs114208039	A/G	−0.23 (−0.52, 0.06)	0.116	−0.05 (−0.19, 0.09)	0.505	−0.05 (−0.17, 0.08)	0.468	−0.06 (−0.23, 0.12)	0.520
	rs73057397	C/T	0 (−0.24, 0.23)	0.975	0.06 (−0.05, 0.18)	0.269	0 (−0.10, 0.10)	0.979	−0.03 (−0.15, 0.10)	0.687
	rs10276619	G/A	0.09 (−0.15, 0.33)	0.440	0.09 (−0.02, 0.21)	0.122	−0.01 (−0.11, 0.09)	0.838	0.06 (−0.06, 0.19)	0.317
	rs445	C/T	−0.11 (−0.36, 0.14)	0.375	0.11 (−0.01, 0.23)	0.061	0.09 (−0.02, 0.19)	0.107	−0.02 (−0.15, 0.11)	0.777
	rs35389394	C/T	−0.12 (−0.37, 0.12)	0.321	−0.01 (−0.13, 0.11)	0.855	0.08 (−0.03, 0.18)	0.156	0.02 (−0.10, 0.15)	0.728
	rs7041895	C/A	0.06 (−0.19, 0.31)	0.651	0.08 (−0.04, 0.2)	0.180	0.01 (−0.10, 0.12)	0.838	−0.09 (−0.22, 0.04)	0.191

Table S5 The associations of total and differential WBC counts related SNPs with confounders in the COW and DFTJ studies.

WBC and subtypes	SNPs	Ref/Alt	Height		Smoking		Alcohol drinking		Exercise	
			β (95% CI)	*P*	β (95% CI)	*P*	β (95% CI)	*P*	β (95% CI)	*P*
	rs10980797	A/G	0.18 (−0.15, 0.51)	0.278	0.02 (−0.14, 0.18)	0.782	−0.11 (−0.25, 0.03)	0.132	0.04 (−0.13, 0.22)	0.629
	rs10987830	A/G	−0.07 (−0.40, 0.26)	0.673	0.05 (−0.11, 0.21)	0.557	−0.16 (−0.30, −0.02)	0.026	−0.07 (−0.24, 0.10)	0.438
	rs687621	A/G	−0.25 (−0.49, −0.01)	0.041	0.02 (−0.10, 0.13)	0.763	0 (−0.10, 0.10)	0.976	−0.04 (−0.16, 0.09)	0.579
	rs6584283	T/C	−0.03 (−0.27, 0.21)	0.818	0.06 (−0.06, 0.17)	0.335	−0.07 (−0.17, 0.03)	0.156	0.03 (−0.10, 0.15)	0.674
	rs75963851	A/G	0.66 (0.09, 1.22)	0.022	−0.09 (−0.37, 0.18)	0.506	−0.03 (−0.27, 0.22)	0.843	0.01 (−0.30, 0.31)	0.972
	rs9551434	A/G	−0.02 (−0.32, 0.28)	0.912	0.03 (−0.11, 0.17)	0.693	0.01 (−0.12, 0.14)	0.906	0.02 (−0.13, 0.18)	0.792
	rs1892548	T/C	−0.11 (−0.37, 0.14)	0.384	−0.06 (−0.18, 0.07)	0.376	0.01 (−0.10, 0.12)	0.923	0.05 (−0.08, 0.18)	0.460
	rs2239630	G/A	0 (−0.24, 0.24)	0.999	0.03 (−0.09, 0.14)	0.639	−0.07 (−0.18, 0.03)	0.158	−0.06 (−0.19, 0.06)	0.325
	rs2004925	A/G	−0.09 (−0.33, 0.14)	0.445	−0.09 (−0.20, 0.03)	0.143	−0.01 (−0.11, 0.09)	0.864	0.01 (−0.11, 0.13)	0.878
	rs12898000	C/G	0.30 (0.06, 0.55)	0.016	−0.08 (−0.20, 0.04)	0.187	−0.02 (−0.13, 0.08)	0.648	−0.08 (−0.21, 0.05)	0.205
	rs57556178	G/A	−0.25 (−0.74, 0.24)	0.324	−0.01 (−0.24, 0.23)	0.951	0.01 (−0.20, 0.22)	0.950	−0.08 (−0.33, 0.17)	0.530
	rs117424492	C/G	−0.10 (−0.36, 0.15)	0.423	0.03 (−0.09, 0.15)	0.604	−0.12 (−0.23, −0.01)	0.031	−0.06 (−0.19, 0.08)	0.392
	rs9674233	T/C	−0.18 (−0.41, 0.06)	0.145	−0.05 (−0.16, 0.06)	0.374	0.02 (−0.08, 0.12)	0.646	0.09 (−0.04, 0.21)	0.166
	rs58681483	A/G	−0.26 (−0.65, 0.13)	0.185	−0.22 (−0.40, −0.03)	0.022	0.09 (−0.08, 0.25)	0.299	−0.09 (−0.29, 0.11)	0.371
	rs12461821	G/A	0.05 (−0.22, 0.32)	0.713	−0.05 (−0.18, 0.07)	0.416	−0.12 (−0.23, 0)	0.044	−0.14 (−0.28, −0.01)	0.042
	rs4811020	C/T	−0.05 (−0.34, 0.25)	0.766	0.11 (−0.03, 0.25)	0.125	−0.05 (−0.17, 0.08)	0.447	0 (−0.15, 0.16)	0.978
Eosinophils										
	rs8191981	G/A	0.09 (−0.45, 0.62)	0.746	0.08 (−0.19, 0.35)	0.573	0.14 (−0.09, 0.38)	0.235	−0.05 (−0.35, 0.26)	0.769
	rs6684992	T/A	0.06 (−0.18, 0.31)	0.616	−0.09 (−0.21, 0.03)	0.136	−0.06 (−0.16, 0.05)	0.270	0.01 (−0.12, 0.13)	0.932
	rs6543119	T/A	0.03 (−0.21, 0.27)	0.805	−0.08 (−0.20, 0.04)	0.181	0.05 (−0.05, 0.15)	0.350	0.04 (−0.08, 0.17)	0.525
	rs13022407	T/C	0.14 (−0.11, 0.39)	0.262	0.04 (−0.08, 0.16)	0.469	−0.02 (−0.13, 0.09)	0.717	−0.01 (−0.14, 0.12)	0.849
	rs35480293	A/C	−0.02 (−0.26, 0.22)	0.852	−0.14 (−0.25, −0.02)	0.020	0.01 (−0.09, 0.12)	0.798	0.04 (−0.09, 0.16)	0.580
	rs7646596	G/T	0.12 (−0.13, 0.37)	0.361	−0.05 (−0.17, 0.07)	0.398	−0.02 (−0.12, 0.09)	0.752	0.03 (−0.10, 0.16)	0.671
	rs2078387	C/A	−0.22 (−0.53, 0.10)	0.185	−0.08 (−0.23, 0.08)	0.330	−0.07 (−0.20, 0.07)	0.318	−0.11 (−0.27, 0.06)	0.214

Table S5 The associations of total and differential WBC counts related SNPs with confounders in the COW and DFTJ studies.

WBC and subtypes	SNPs	Ref/Alt	Height		Smoking		Alcohol drinking		Exercise	
			β (95% CI)	*P*	β (95% CI)	*P*	β (95% CI)	*P*	β (95% CI)	*P*
	rs114965247	T/G	−0.22 (−0.59, 0.15)	0.236	0.03 (−0.15, 0.21)	0.763	0.08 (−0.07, 0.24)	0.300	−0.02 (−0.25, 0.21)	0.865
	rs2210366	A/G	0.33 (0.09, 0.57)	0.008	0.02 (−0.10, 0.14)	0.737	−0.03 (−0.14, 0.07)	0.553	0.05 (−0.08, 0.17)	0.456
	rs9986877	T/A	0.01 (−0.26, 0.28)	0.958	0.02 (−0.11, 0.15)	0.744	−0.02 (−0.14, 0.09)	0.724	−0.02 (−0.16, 0.11)	0.730
	rs445	C/T	−0.11 (−0.36, 0.14)	0.375	0.11 (−0.01, 0.23)	0.061	0.09 (−0.02, 0.19)	0.107	−0.02 (−0.15, 0.11)	0.777
	rs6994642	C/T	−0.03 (−0.37, 0.31)	0.864	−0.16 (−0.33, 0)	0.051	−0.04 (−0.19, 0.11)	0.589	−0.02 (−0.19, 0.16)	0.867
	rs4562281*	G/C	−0.15 (−0.45, 0.15)	0.332	−0.05 (−0.20, 0.09)	0.461	**−0.19 (−0.32, −0.06)**	**0.003**	−0.10 (−0.26, 0.06)	0.213
	rs16917546	T/C	−0.01 (−0.26, 0.24)	0.934	0.08 (−0.04, 0.20)	0.177	−0.03 (−0.13, 0.08)	0.624	0.10 (−0.03, 0.23)	0.122
	rs7114362	T/C	−0.01 (−0.25, 0.23)	0.931	0.01 (−0.11, 0.12)	0.904	0.03 (−0.07, 0.13)	0.579	0.08 (−0.05, 0.20)	0.238
	rs117072053	T/C	−0.11 (−0.36, 0.15)	0.406	0.03 (−0.09, 0.15)	0.606	−0.12 (−0.22, −0.01)	0.035	−0.06 (−0.19, 0.08)	0.397
	rs9901392	C/T	0.20 (−0.12, 0.51)	0.215	−0.19 (−0.34, −0.03)	0.017	−0.05 (−0.18, 0.09)	0.494	0.11 (−0.04, 0.27)	0.156
	rs7249415	A/C	−0.23 (−0.48, 0.01)	0.062	−0.01 (−0.13, 0.10)	0.824	0.01 (−0.09, 0.12)	0.832	0 (−0.13, 0.12)	0.968
Basophils										
	rs6703781	G/A	−0.08 (−0.33, 0.17)	0.536	0.10 (−0.02, 0.22)	0.098	0.07 (−0.03, 0.17)	0.172	−0.04 (−0.22, 0.13)	0.639
	rs6427756	A/G	0.11 (−0.19, 0.42)	0.469	0.04 (−0.19, 0.27)	0.750	0.04 (−0.18, 0.25)	0.732	−0.03 (−0.19, 0.14)	0.727
	rs4951254	C/T	−0.25 (−0.48, −0.01)	0.041	−0.08 (−0.28, 0.11)	0.401	0.10 (−0.08, 0.27)	0.269	0.07 (−0.10, 0.23)	0.441
	rs16823866	T/C	0.04 (−0.26, 0.34)	0.788	0.17 (−0.07, 0.42)	0.165	0.18 (−0.05, 0.41)	0.123	0.14 (−0.06, 0.34)	0.178
	rs3770136	C/T	0.09 (−0.21, 0.38)	0.571	0.02 (−0.18, 0.21)	0.866	−0.10 (−0.32, 0.13)	0.405	0.15 (−0.07, 0.37)	0.167
	rs3804785	G/T	0.07 (−0.19, 0.33)	0.574	−0.09 (−0.20, 0.03)	0.126	0.14 (0.04, 0.25)	0.009	0 (−0.18, 0.18)	0.983
	rs6782812	A/G	0.12 (−0.13, 0.37)	0.358	−0.07 (−0.18, 0.04)	0.224	−0.03 (−0.22, 0.15)	0.721	0.11 (−0.07, 0.28)	0.221
	rs73069193	A/G	0.31 (−0.31, 0.93)	0.322	0.15 (−0.26, 0.56)	0.471	−0.22 (−0.57, 0.14)	0.241	0.12 (−0.33, 0.57)	0.605
	rs1408	G/A	−0.01 (−0.25, 0.22)	0.913	−0.11 (−0.21, −0.01)	0.029	−0.08 (−0.25, 0.09)	0.368	0.12 (−0.04, 0.29)	0.144
	rs113721499	C/T	−0.21 (−0.51, 0.08)	0.157	−0.10 (−0.46, 0.26)	0.601	−0.11 (−0.29, 0.07)	0.235	−0.02 (−1.59, 1.54)	0.976
	rs12208785	G/A	−0.07 (−0.37, 0.24)	0.675	−0.08 (−0.33, 0.18)	0.553	0.05 (−0.18, 0.28)	0.675	0.20 (0, 0.40)	0.051
	rs7008536	C/A	−0.12 (−0.36, 0.12)	0.320	0.04 (−0.16, 0.24)	0.688	−0.17 (−0.34, 0.01)	0.062	0.13 (0.03, 0.23)	0.008

Table S5 The associations of total and differential WBC counts related SNPs with confounders in the COW and DFTJ studies.

WBC and subtypes	SNPs	Ref/Alt	Height		Smoking		Alcohol drinking		Exercise	
			β (95% CI)	*P*	β (95% CI)	*P*	β (95% CI)	*P*	β (95% CI)	*P*
	rs2980884	A/G	−0.05 (−0.31, 0.21)	0.698	0.13 (−0.09, 0.34)	0.245	−0.02 (−0.16, 0.13)	0.823	−0.10 (−0.29, 0.09)	0.317
	rs10100356*	A/G	0 (−0.23, 0.23)	0.997	0.10 (−0.10, 0.29)	0.324	**0.16 (0.13, 0.18)**	**<0.001**	0.06 (−0.11, 0.22)	0.478
	rs741804†	A/C	−0.03 (−0.36, 0.30)	0.860	0.11 (−0.10, 0.33)	0.299	−0.07 (−0.32, 0.18)	0.587	0.03 (−0.21, 0.26)	0.816
	rs150876292†	T/C	0.51 (−0.51, 1.52)	0.331	0.16 (−0.50, 0.81)	0.640	0.12 (−0.50, 0.74)	0.704	−0.37 (−1.04, 0.31)	0.287
	rs11018874	G/A	−0.07 (−0.34, 0.20)	0.609	0.02 (−0.20, 0.24)	0.853	−0.07 (−0.21, 0.07)	0.315	−0.01 (−0.2, 0.17)	0.890
	rs695113	T/C	−0.09 (−0.34, 0.17)	0.500	−0.07 (−0.21, 0.08)	0.368	−0.16 (−0.36, 0.03)	0.095	0.12 (−0.07, 0.31)	0.204
	rs7315361	A/T	−0.18 (−0.42, 0.06)	0.141	0 (−0.19, 0.19)	0.998	0.15 (−0.02, 0.33)	0.091	−0.12 (−0.2, −0.05)	0.002
	rs61123801	G/A	−0.10 (−0.38, 0.18)	0.489	0.02 (−0.21, 0.25)	0.885	0.06 (−0.15, 0.27)	0.584	−0.15 (−0.35, 0.05)	0.138
	rs1474920	T/G	−0.06 (−0.31, 0.19)	0.644	0.02 (−0.18, 0.21)	0.861	0.03 (−0.15, 0.20)	0.761	0.12 (−0.02, 0.25)	0.085
	rs11857230	A/T	0.02 (−0.24, 0.27)	0.899	0.06 (−0.15, 0.27)	0.596	0.02 (−0.17, 0.21)	0.806	−0.01 (−0.18, 0.16)	0.931
	rs12445547†	T/G	−0.23 (−0.46, 0.01)	0.060	0.01 (−0.19, 0.21)	0.905	0.13 (−0.05, 0.31)	0.155	**−0.17 (−0.23, −0.11)**	**<0.001**
	rs7253959†	A/G	−0.06 (−0.29, 0.18)	0.643	−0.07 (−0.25, 0.12)	0.498	0.09 (−0.08, 0.26)	0.305	−0.13 (−0.29, 0.04)	0.142
	rs28530618	G/A	0.02 (−0.23, 0.28)	0.860	−0.09 (−0.29, 0.11)	0.367	0.08 (−0.10, 0.26)	0.387	−0.06 (−0.23, 0.12)	0.537
	rs78762153	C/T	−0.05 (−0.45, 0.35)	0.809	−0.01 (−0.32, 0.30)	0.958	−0.10 (−0.36, 0.18)	0.490	0.19 (−0.08, 0.46)	0.164

Abbreviations: WBC, white blood cell; COW, coke-oven workers cohort; DFTJ, Dongfeng-Tongji cohort.

Notes: Effect estimates were obtained from linear regression model for height and logistic regression model for smoking, drinking and exercise, with adjustment for age, gender, and chip types (only in DFTJ study). Fixed-effect (heterogeneity $P \geq 0.05$) or random-effect (heterogeneity $P < 0.05$) meta-analysis was used to combine results from COW and DFTJ studies (n=4,012).

*These SNPs were significantly associated with the covariates at the Bonferroni-corrected level and they were then excluded as IVs in the following MR analysis.

†These SNPs were identified as potential outliers by MR-PRESSO method and they were further excluded as IVs in the following MR analysis.

Table S6 Between-instrument heterogeneity test for the Mendelian randomization analyses of total and differential WBC counts with lung function.

Total and differential WBC counts	No. of SNPs	MR Method	FVC, mL		FEV_1, mL	
			Cochran's Q	Heterogeneity *P*	Cochran's Q	Heterogeneity *P*
**Total WBC**	33	IVW	24.74	0.816	27.56	0.691
		MR-Egger	23.64	0.825	26.10	0.716
**Neutrophils**	19	IVW	18.55	0.420	20.37	0.312
		MR-Egger	17.30	0.434	18.46	0.361
**Monocytes**	30	IVW	27.24	0.559	23.44	0.756
		MR-Egger	26.59	0.541	22.60	0.753
**Eosinophils**	17	IVW	18.50	0.295	13.97	0.601
		MR-Egger	18.36	0.244	13.79	0.541
**Basophils**	21	IVW	30.49	0.062	26.67	0.145
		MR-Egger	30.03	0.051	26.18	0.125

Abbreviations: WBC, white blood cell; FVC, forced vital capacity; FEV_1, forced expiratory volume in one second; IVW, inverse-variance weighted; MR, Mendelian randomization.

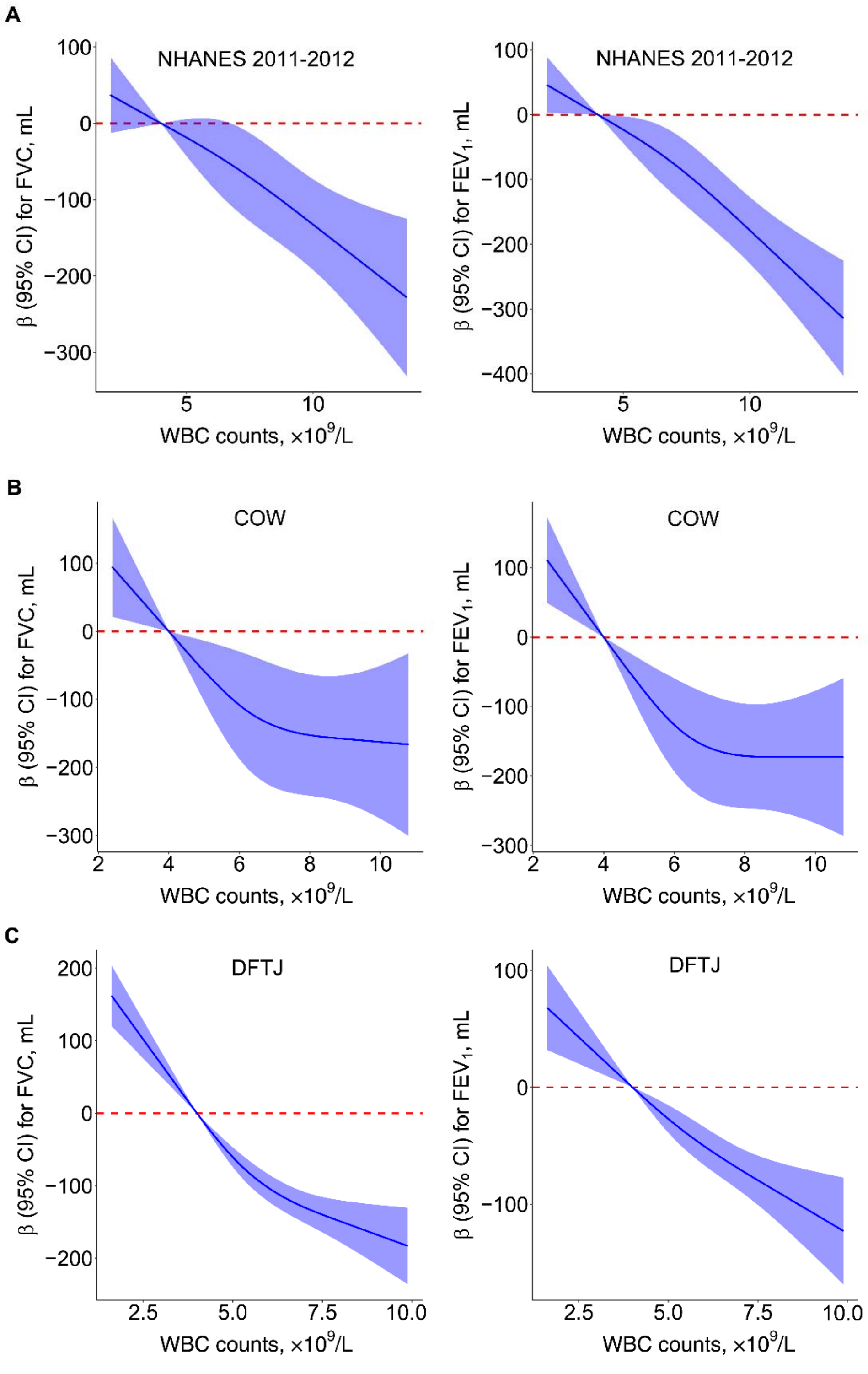

Figure S1 Restricted cubic spline plot to show the relationship of total WBC counts with lung function.

Abbreviations: FVC, forced vital capacity; FEV_1, forced expiratory volume in one second; NHANES 2011-2012, National Health and Nutrition Examination Survey 2011-2012; WBC, white blood cell; COW, coke-oven workers cohort; DFTJ, Dongfeng-Tongji cohort.

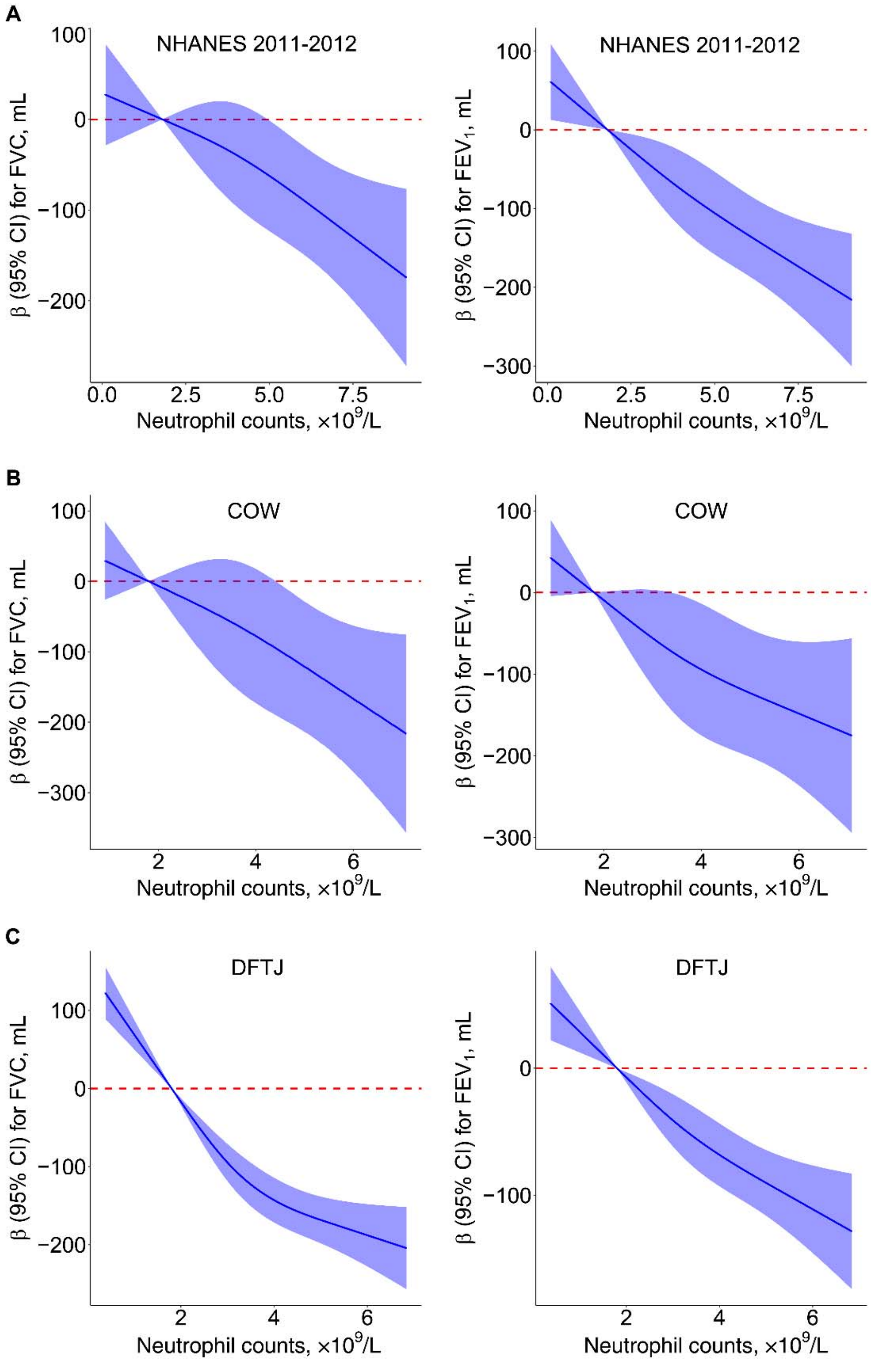

Figure S2 Restricted cubic spline plot to show the relationship of neutrophil counts with lung function.

Abbreviations: FVC, forced vital capacity; FEV_1, forced expiratory volume in one second; NHANES 2011-2012, National Health and Nutrition Examination Survey 2011-2012; COW, coke-oven workers cohort; DFTJ, Dongfeng-Tongji cohort.

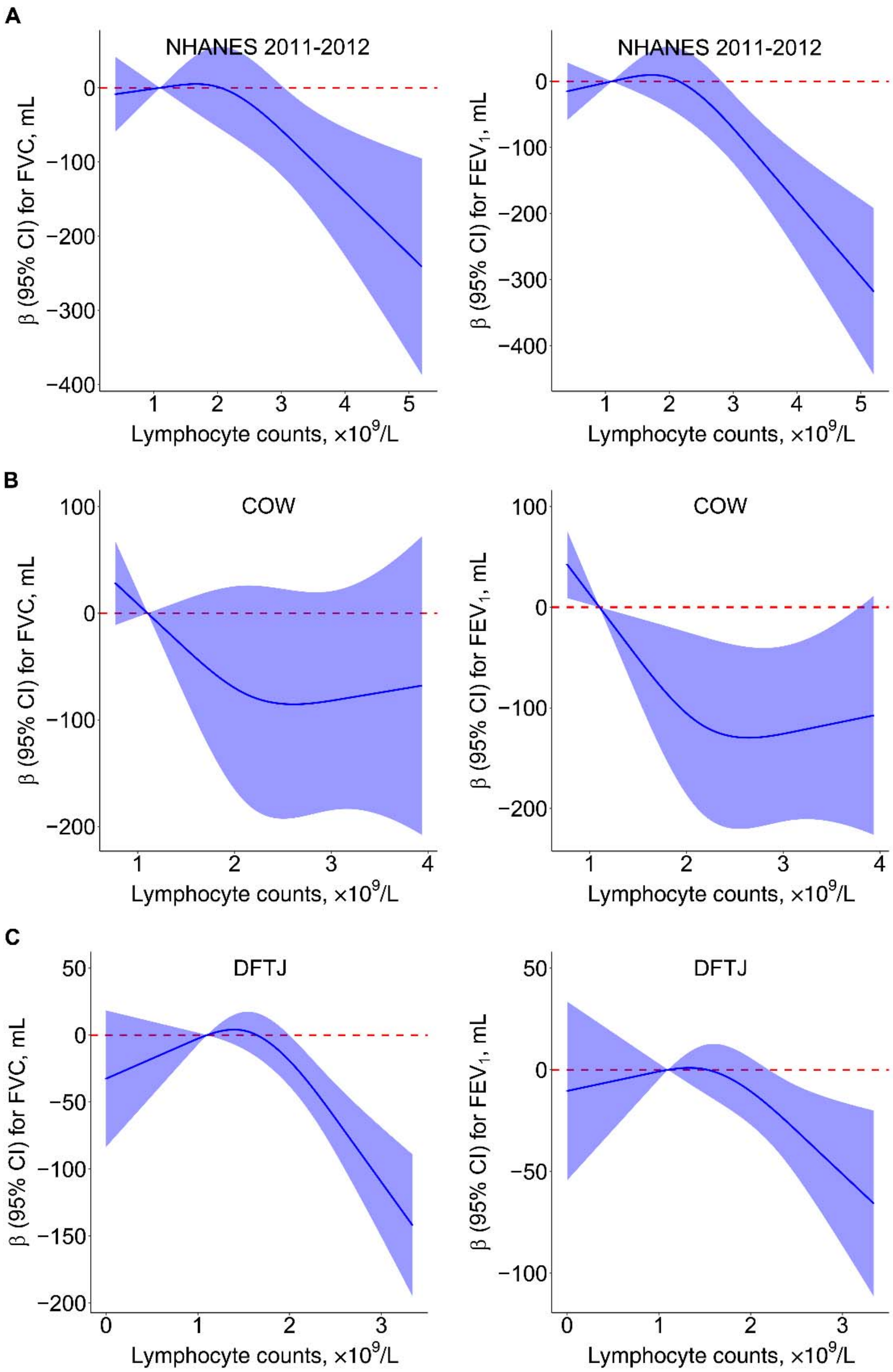

Figure S3 Restricted cubic spline plot to show the relationship of lymphocyte counts with lung function.

Abbreviations: FVC, forced vital capacity; FEV_1, forced expiratory volume in one second; NHANES 2011-2012, National Health and Nutrition Examination Survey 2011-2012; COW, coke-oven workers cohort; DFTJ, Dongfeng-Tongji cohort.

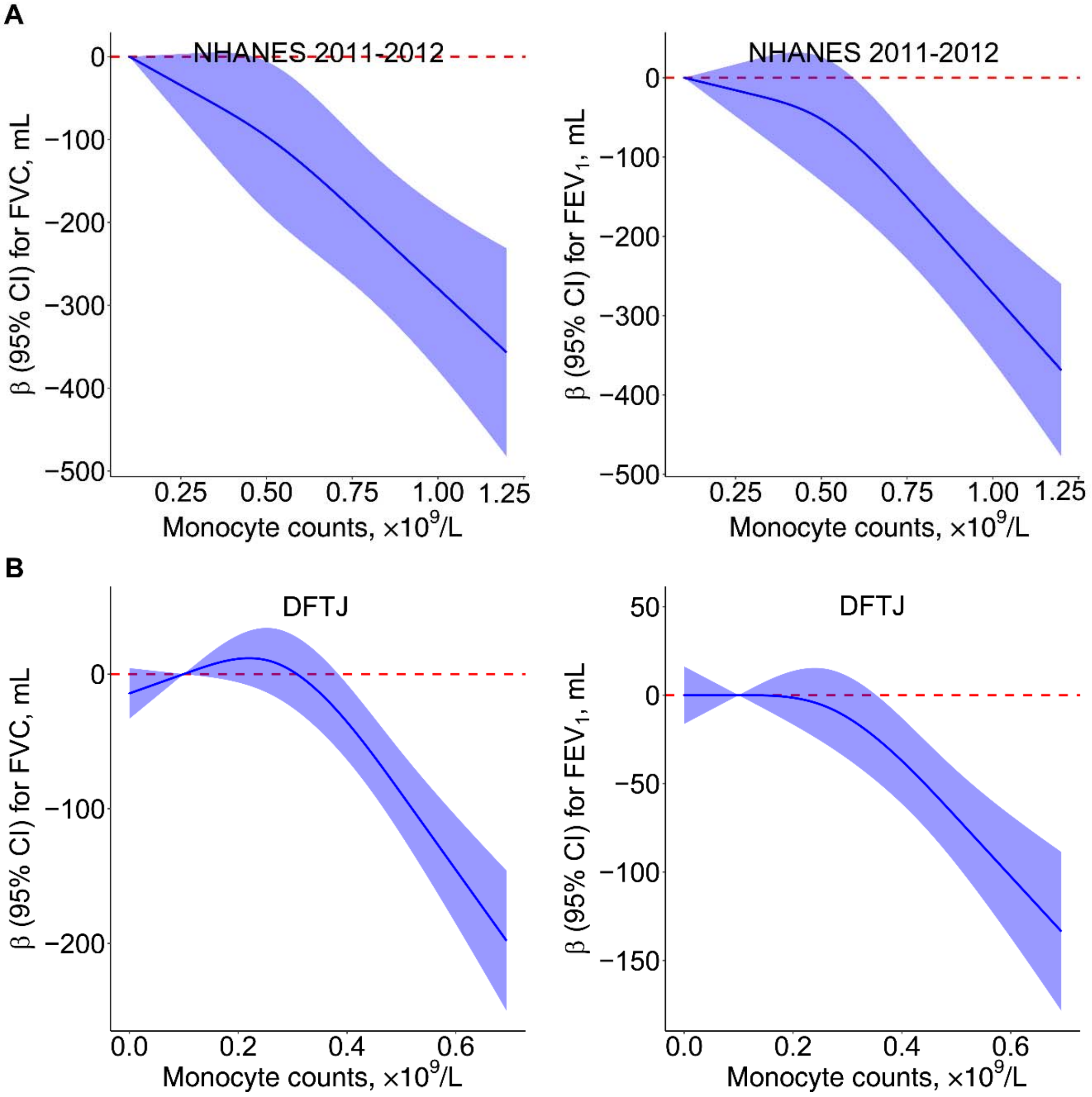

Figure S4 Restricted cubic spline plot to show the relationship of monocyte counts with lung function.

Abbreviations: FVC, forced vital capacity; FEV_1, forced expiratory volume in one second; NHANES 2011-2012, National Health and Nutrition Examination Survey 2011-2012; DFTJ, Dongfeng-Tongji cohort.

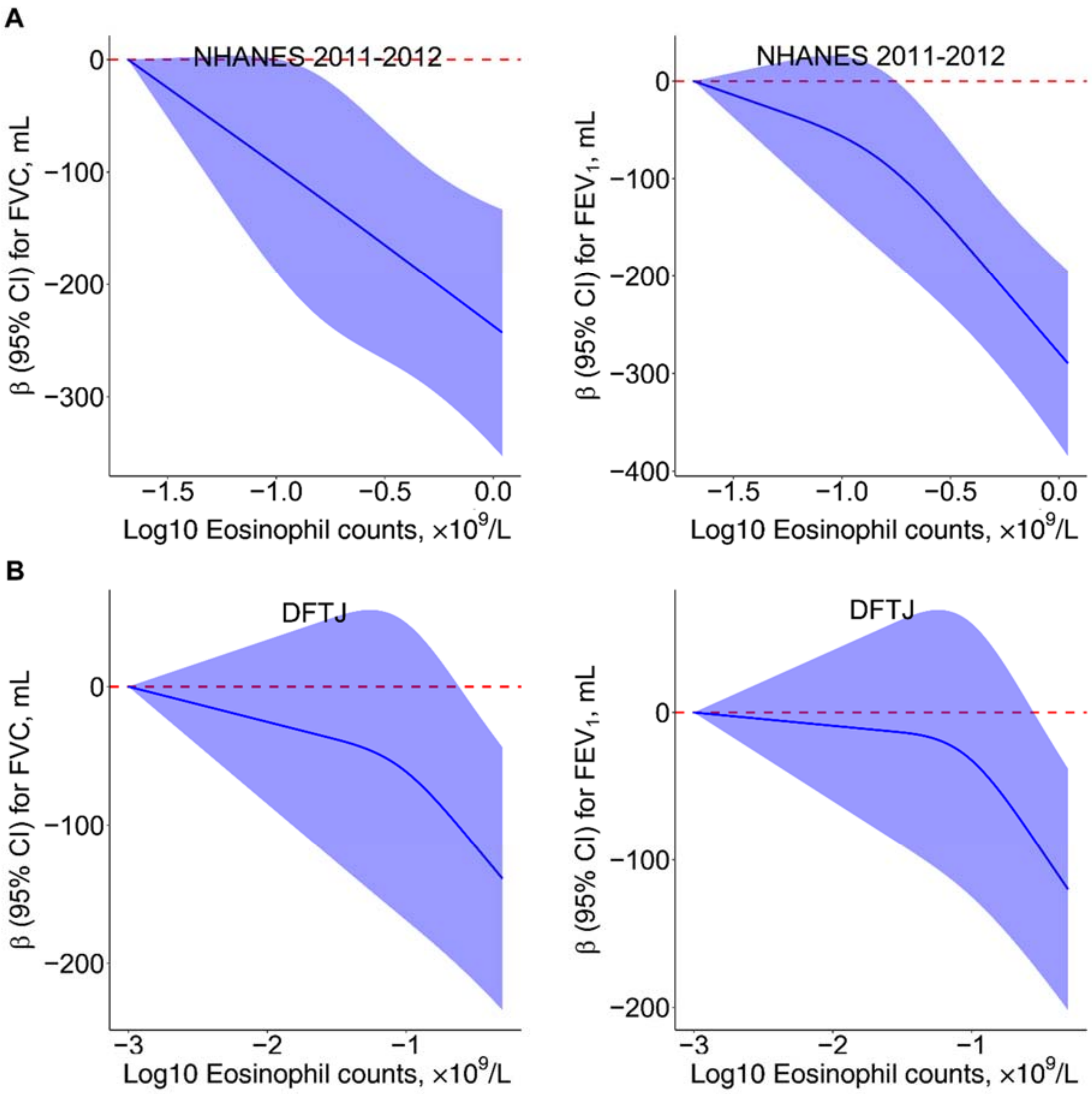

Figure S5 Restricted cubic spline plot to show the relationship of eosinophil counts with lung function.

Abbreviations: FVC, forced vital capacity; FEV_1, forced expiratory volume in one second; NHANES 2011-2012, National Health and Nutrition Examination Survey 2011-2012; DFTJ, Dongfeng-Tongji cohort.

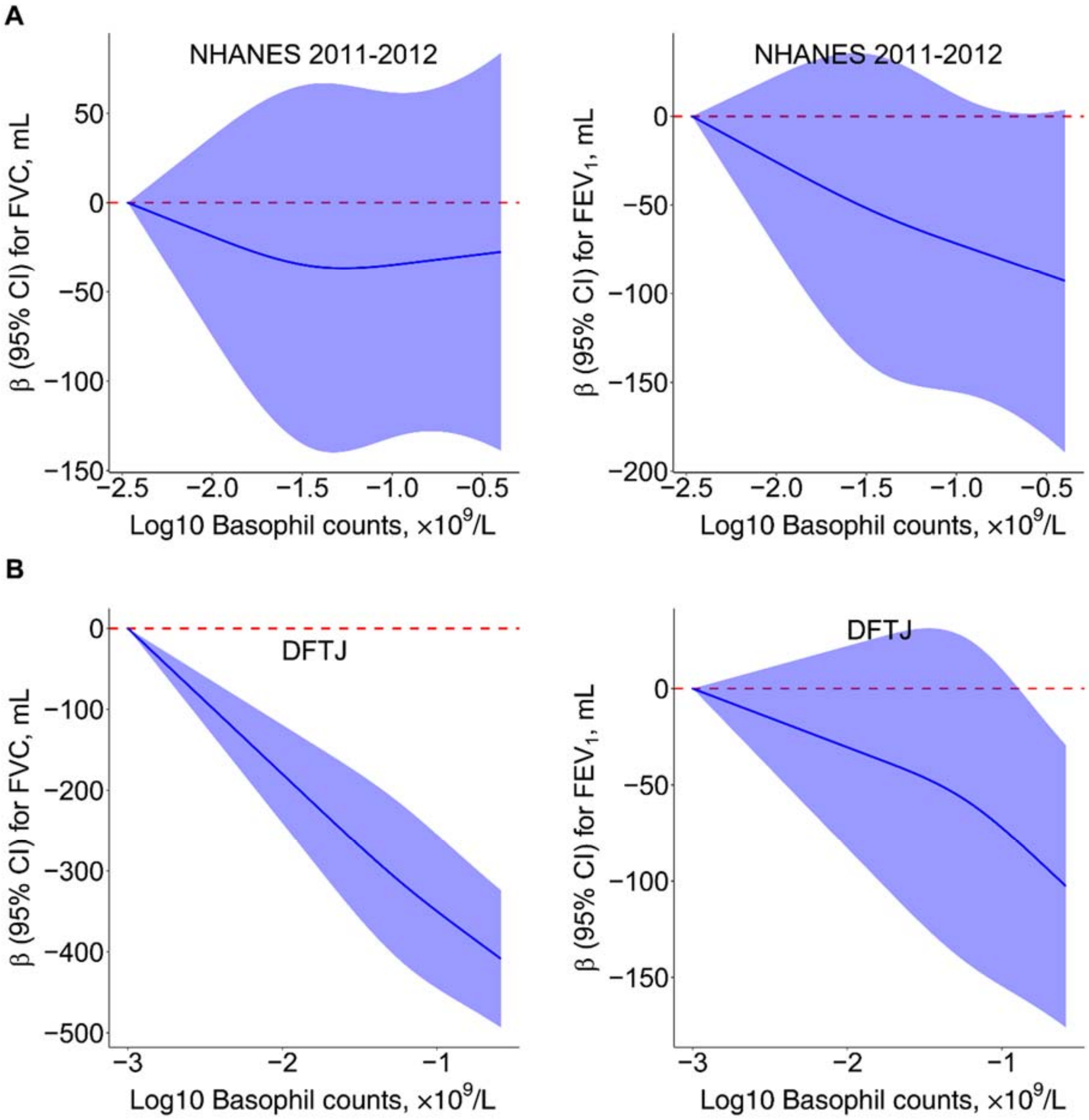

Figure S6 Restricted cubic spline plot to show the relationship of basophil counts with lung function.

Abbreviations: FVC, forced vital capacity; FEV_1, forced expiratory volume in one second; NHANES 2011-2012, National Health and Nutrition Examination Survey 2011-2012; DFTJ, Dongfeng-Tongji cohort.

References

[1] Flegal KM, Kruszon-Moran D, Carroll MD, et al. Trends in Obesity Among Adults in the United States, 2005 to 2014. JAMA. 2016;315(21):2284-2291.

[2] Taylor AL, Denniston MM, Klevens RM, et al. Association of Hepatitis C Virus With Alcohol Use Among U.S. Adults: NHANES 2003-2010. Am J Prev Med. 2016;51(2):206-215.

[3] Zhao G, Li C, Ford ES, et al. Leisure-time aerobic physical activity, muscle-strengthening activity and mortality risks among US adults: the NHANES linked mortality study. Br J Sports Med. 2014;48(3):244-249.

[4] Lai X, Yang L, Légaré S, et al. Dose-response relationship between serum uric acid levels and risk of incident coronary heart disease in the Dongfeng-Tongji Cohort. Int J Cardiol. 2016;224:299-304.

[5] Kanai M, Akiyama M, Takahashi A, et al. Genetic analysis of quantitative traits in the Japanese population links cell types to complex human diseases. Nat Genet. 2018;50(3):390-400.
